# Recent Advances in Internet of Things (IoT) Infrastructures for Building Energy Systems: A Review †

**DOI:** 10.3390/s21062152

**Published:** 2021-03-19

**Authors:** Wahiba Yaïci, Karthik Krishnamurthy, Evgueniy Entchev, Michela Longo

**Affiliations:** 1 CanmetENERGY Research Centre, Natural Resources Canada, 1 Haanel Drive, Ottawa, ON K1A 1M1, Canada; evgueniy.entchev@canada.ca; 2 Candela IoT, Mountain View, CA 94043, USA; karkrish2009@gmail.com; 3 Department of Energy, Politecnico di Milano, via La Masa, 20156 Milan, Italy; michela.longo@polimi.it

**Keywords:** Internet of Things (IoT), building energy management, smart energy systems, energy efficiency, energy control

## Abstract

This paper summarises a literature review on the applications of Internet of Things (IoT) with the aim of enhancing building energy use and reducing greenhouse gas emissions (GHGs). A detailed assessment of contemporary practical reviews and works was conducted to understand how different IoT systems and technologies are being developed to increase energy efficiencies in both residential and commercial buildings. Most of the reviewed works were invariably related to the dilemma of efficient heating systems in buildings. Several features of the central components of IoT, namely, the hardware and software needed for building controls, are analysed. Common design factors across the many IoT systems comprise the selection of sensors and actuators and their powering techniques, control strategies for collecting information and activating appliances, monitoring of actual data to forecast prospect energy consumption and communication methods amongst IoT components. Some building energy applications using IoT are provided. It was found that each application presented has the potential for significant energy reduction and user comfort improvement. This is confirmed in two case studies summarised, which report the energy savings resulting from implementing IoT systems. Results revealed that a few elements are user-specific that need to be considered in the decision processes. Last, based on the studies reviewed, a few aspects of prospective research were recommended.

## 1. Introduction

**Figure 1:**
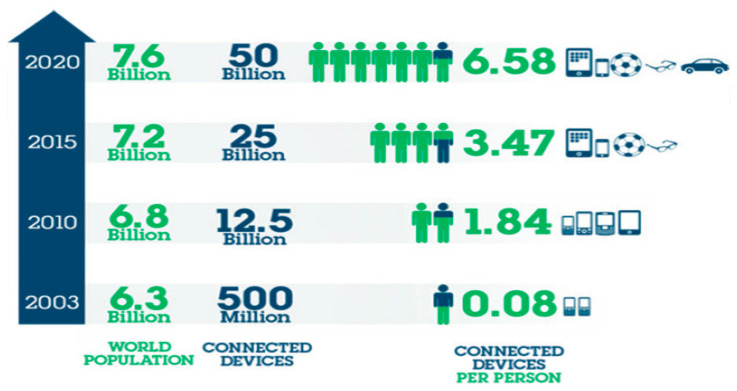
The overall world population and the connected devices by 2020 [6].

The Internet of Things (IoT) is a relatively new and rapidly growing technology. It began in the early 2000s where RFID technology was used for inventory management and product delivery tracking [1]. The IoT is a system of interconnected computing machines, which are utilised to automate (and thus simplify) a variety of aspects of routine human living and assist in creating informed approach evaluations to warrant that diverse activities are implemented in the best resource effective approaches possible. While the notion of IoT has been widespread in academic communities since the late nineties, developments are yet in their early stages [2]. The total of connected devices around the world is projected to be in tens of billions. Therefore, as the number of connected devices worldwide is expected to continue to expand exponentially, IoT systems are anticipated to become much more prevalent in the coming years [3,4,5]. Since this beginning, the number of connected devices has been ever increasing to an estimated 50 billion devices by 2020, shown below in Figure 1 [6]. The number of different applications of IoT has been the main reason for the constant growth. IoT is proving to be useful in countless applications, collecting data that was once collected manually or never collected. These data are used to add convenience and safety to our lives, as well as increase profit and efficiencies of companies.

From an architectural perspective, IoT systems connect various sensors and smart devices to a local or cloud-based controller. Sensors often collect and transmit real-time data about their environment. This information is then used by controllers to offer both immediate and long-term responses. Predictive and adaptive algorithms can help controllers execute operational responses from the simple to complex. A simple implementation could be the turning on of the lights in an office space based on signals from in-room occupancy sensors. The complex may optimise an entire heating, ventilation and air conditioning (HVAC) system based on a long-term understanding of occupancy and climatic patterns for that office space.

IoT systems are somewhat reliable in their architecture and consist of three primary layers: (i) the hardware or sensor layer, (ii) the software control layer and (iii) the application layer. The hardware or sensor layer includes physical components with unique identifiers and the capacity to transmit information over a network without necessitating human-to-human or human-to-computer exchanges. Instances comprise a network of remote sensors to monitor and transfer data on environmental conditions, or a collection of actuators to control a building’s heating or lighting appliances. Several of today’s appliances are “smart”, in that they expressively interchange information amongst each other. The size and scope of today’s IoT systems also differ; they can range from a small number of sensors in a residential building, to millions of gadgets on complex factory floors and within large commercial buildings [7]. When these assemblies of machines are connected, robust software layers must be designed in order to enable and control communications amongst these device congregations or assemblies and execute any needed actuation (for instance, the sending of a signal to “turn on” a furnace). Particular software controls in turn rely on the particular applications for which the IoT systems were designed.

**Figure 2:**
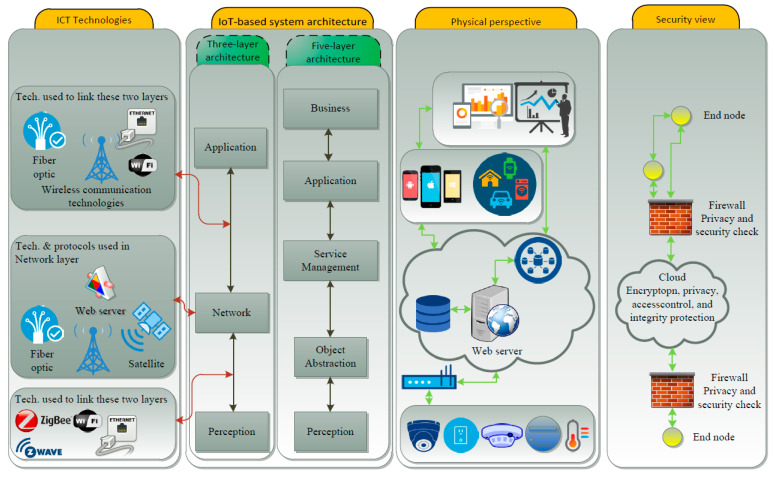
Internet of Things (IoT)-based system architecture [8].

IoT systems should enhance the applications and use cases for which they are implemented. As with any good engineering practice, end users drive given design choices. A not so unique twist is that customisation of IoT systems for human behaviour habitually translate into the recurrent collection and transmission of data pertaining to human activity and behaviour profiles that in turn might be intrusive to user activities. It follows that, privacy and security of customers are critical and sensitive factors that need to be taken into consideration in order for an IoT system to be sustainable in the long term and on a large scale. Figure 2 shows an illustration for the different modules required for an operational IoT system [8].

Building energy management offers its specific series of distinctive constraints and possible solutions. The notion of a “smart building” is getting progressively more widespread. A smart building is one, which is supplied with modern technologies to automate procedures, particularly HVAC controls, and contain energy consumption, for example, wall outlets using smart technologies. Although inhabitant convenience is an essential concern, the main objective is to optimise a building’s energy effectiveness, decreasing losses and warranting that energy is only utilised when it is required.

Smart buildings exploit distinct datasets to define how diverse smart technologies will work both separately and in combination with one another. The disparate datasets include both historical data, such as energy usage profiles and human occupancy patterns, and real-time data, such as current temperature conditions and current human occupancy of a room. A sample arrangement of smart location-based energy controls that change current static and centralised energy controls into dynamic and distributed energy controls is shown in [9]. The IoT architecture comprises four components: monitoring and control via mobile devices, location-based automatic controls, cloud-computing platform for data storage and computation and applications that implement proven energy saving policies [9,10].

As indicated in the literature, several reviews have been completed on IoT systems [7,8,9,10,11,12,13,14,15,16]. The interpretation deduced is that it seems that no survey has been accomplished on IoT infrastructures that are specific to building energy systems. The present review is thus an original research initiative albeit built upon previous studies [17,18]. This review paper does not retrace the enormous academic and industry literature that has already been established around industrial IoT solutions. Instead, it focuses on a narrower but deeper analysis of current thinking in the area of IoT systems for building energy applications.

The motivation for this survey is threefold. First, building energy IoT represents a significant and “easier to attain” solution for supporting global sustainability. As has been well documented by Energy Information Administration (EIA) and other industry research, over a third (if not more) of all energy is consumed within commercial and residential buildings. Even a small percentage improvement in energy consumption or reduction of wastages could therefore yield substantial environmental and financial benefits. Second, building energy IoT systems are driven by the domain-specific vision of “smart buildings” that is distinct enough from the end-state visions of more prevalent industrial IoT solutions to warrant solutions that either unique to or customised specifically for buildings. “Smart buildings” use a networked architecture of sensors/actuators that are directly connected to different building subsystems (such as lighting, HVAC, elevators and surveillance). Adaptive and predictive controllers and associated control algorithms can then reduce energy consumption, reduce wastage and improve user/occupant experiences. Third, and finally, there are significant complexities in building energy environments arising from (i) a large variety of disparate datasets and (ii) different controller configurations. As additional context, for the second driver, controller configurations can be either centralised or decentralised. As common with most centralised architectures, centralised building controls are more efficient but come with a single point of failure. Decentralised or distributed architectures are, on the other hand, more complex to execute, often requiring significant coordination and communications among the distributed controlling mechanisms. However, on the flip side, these distributed architectures offer better risk mitigation.

**Figure 3:**
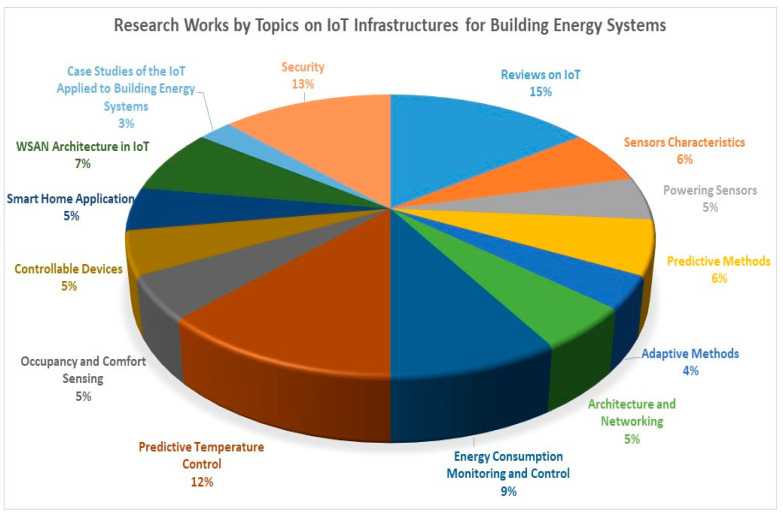
Research works by topics on IoT infrastructures for building energy systems.

Figure 3 and Table 1 provides the summary of research works on IoT infrastructures in building energy systems.

The survey is covered in Section 2, Section 3, Section 4, Section 5, Section 6, Section 7 and Section 8 as follows: Section 2 confirms that IoT solutions in fact offer feasible and potentially high impact solutions, then it drills down into sensors, especially the issue of powering sensors. Building IoT solutions requires a large number of low-power sensors. Getting sustained power to these sensors in a scalable and reliable way is one of the big bottlenecks for the commercial expansion of building IoT. Section 3 discusses different controller methods. Essentially control methods are either predictive or adaptive. Predictive methods use a variety of statistical techniques on historical data, such as weather or occupancy, to discern patterns and predict possible values for the future planning. Adaptive methods are more intrusive in that they actively impact the settings of building controls and operations. More recent innovations combine both predictive and adaptive controls into hybrid methods. In Section 4, the application of traditional IoT networking solutions for building energy IoT solutions are discussed. The good news is that many of the emerging IoT networking protocols remain applicable in building contexts. Section 5 provides some case studies of the IoT applied to building energy systems. In Section 6, attention is devoted to security solutions for Building IoT systems. As with any distributed IoT system, security is critical for building IoT systems as well. The challenge in both commercial and residential building contexts is that users are often less sophisticated and sometimes lack a good understanding on the different types of threats, such as denial of service or sleep deprivation attacks, and their possible impact, such as inference of occupancy patterns and building usage from building operations data. Furthermore, from a technical perspective, building IoT controllers often have low computing power to support sophisticated security and data encryption algorithms. Security in building energy IoT will be about making “liveable” compromises between system capabilities and security aspirations. The limitations and challenges involved with the IoT are summarised in Section 7. Finally, the last section, Section 8, concludes with an outline of main understandings and recommended directions for further research.

## 2. Sensors Characteristics

This section gives a summary of types of sensors and powering of sensors.

### 2.1. Sorts of Sensors

A number of researchers have been working on validating the application of IoT systems for achieving tangible energy savings.

Casini [19] has assessed the feasibility of a few smart technologies for realising energy efficiencies, including intelligent controls of HVAC, home lighting, high-power appliances, and hot water circulation. Specifically, his study has demonstrated that each of these processes are capable of remote actuation and can thus be applied in an IoT energy management system.

Wicaksono et al. [20] and Lork et al. [21] have further corroborated the feasibility of these smart technologies for home energy management.

King and Perry [22] have taken this analysis a step further. They have quantified the likely ranges of energy savings that are achievable for a selection of residential IoT sensors. The sensors that they studied varied greatly in both function and convolution, assorting from proximity sensors that transfer discrete Boolean numbers, to air quality sensors that collect diverse sets of data points and transmit them as analogue numbers. When these sensors work in arrangement with other smart technologies, they can extend from increasing the energy efficiency of a single home process to deploying a smart building with energy autonomy. The range of realised energy savings also varies. Table 2 [22] below summarises the diverse sensors, associated smart technologies and the savings they could realise. For instance, an upgrade of a single aspect of home energy use could possibly save 5–15% of power that would otherwise be needed, while a smart building with such energy-autonomy could realise savings of up to 50%.

Png et al. [23] have taken an orthogonal approach to powering sensors by dramatically reducing power requirements for building IoT systems. They investigated an application in which several sensors for analysing air quality can be combined within a single Arduino component for control and data processing. These sensors range amid only analog sensors integrated with the Arduino’s analog I/O pins, discrete Boolean state signals and digital empirical signals. When the system is integrated by means of a low power controller, and power is supplied between sensors over pins on the controller, the power draw of the system can be only 100 mW at full power.

### 2.2. Powering Sensors

The primary challenge in deploying sensors for residential and commercial energy management systems are related to power distribution to these sensors and their lifecycle management. Most building IoT sensors require very little power especially when compared to the energy consumption of the devices that they control. Yet, the actual distribution of power to these dispersed sensors may quickly come out to be problematic, given the large numbers of sensors that may often be deployed across the home or building. While single-cell battery technologies are habitually sufficient to power these devices, maintenance and replacement of these battery power sources become tedious. In this section, we explore some of the alternative solutions that have been proposed to power these distributed sensors.

King and Perry [22] have explored the use of Power over Ethernet (PoE) for powering sensors in a smart lighting application. PoE provided the dual advantage of both provisioning power and offering a channel for communications between sensors and controllers. The recommended utilisation powered both the sensors and the lights with PoE, streamlining the electrical layout of the entire system. A drawback of this method, however, is the need for hardwired connectivity between peripheral power sources and connected sensors. A further drawback is that PoE cannot supply more than 57V DC, which is insufficient to power current lighting systems. PoE is therefore not ideal when considering improvements to existing energy systems, but may offer a convenient approach to deploying new applications in existing environments.

Prauzek et al. [24] have studied the ability to adapt traditional large-scale renewable energy sources (wind, solar, etc.) for milliwatt-level power required by building sensors. They examined processes for extracting energy on this small scale, comprising the trade-offs between micro turbine size and reliability in an outdoor environment. Their study showed that these technologies could produce up to 10 mW of power under favourable settings. They also appraised some energy management procedures, where excess power produced at the sensor levels can be diverted to the core controllers as necessary. Their outcomes are also corroborated by the work of Gawali and Deshmukh [25].

An equivalent study was conducted by Curry and Harris [26] who assessed a number of renewable sources to power building IoT sensors. Table 3 summarises their findings.

## 3. Control Methods

This section gives an outline of IoT predictive and adaptive methods.

### 3.1. Predictive Methods

Predictive control methods are mainly designed to forecast energy use using a number of parameters including historical energy utilisation profiles, local weather and human behaviours. A key insight for building predictive algorithms is that while instant energy use or external weather can vary, broad tendencies in historical data remain mostly steady and predictable. Residential occupancy measured over several days could, for instance, indicate typical occupancy patterns of a household or even those of individuals within that household. While these occupancy patterns may vary on any given day, studies show that they often hold to within 10% of the general trend [22]. In fact, such time-based analysis can be extended over longer periods of time. For example, it is intuitive to assume that a house occupant who works from Monday to Friday and spends weekends at home would exhibit similar energy use patterns over several weeks, and that these patterns will also likely exhibit seasonal variations through the course of a year. In addition, households within core working years may even exhibit similar patterns over the course of multiple years.

A comparable study can be carried out over a time scale of months or years. Data pertaining to the climate can be collated through a long duration in order to forecast weather patterns on a longer term. While it is difficult to predict weather conditions, the weekly or monthly trends collected over a few years can be more easily retrieved and analysed and averages derived as a baseline for future predictions. As Matsui et al. [27] suggested, it is valuable to have average historical weather data statistics as a reference for analyses.

Various approaches could be utilised to analyse and combine data for the purpose of minimising errors and outliers. These methods include advanced statistical modelling approaches such as multiple linear regressions, adaptive linear filtering, normalised least means square, recursive least square and the Gaussian mixture models. The efficiency of every single of these methods differs, and depends on the application utilised [28,29].

Information resultant with an accurate data set could be utilised to develop energy control algorithms as examined by Mataloto et al. [30]. In the application of heating and cooling especially, historical data could be exploited to establish temperature (and energy consumption) standards, which the system must attempt to maintain. This might fluctuate though, subject to specific environments. Usually, a dead zone of 4–6 °C is used; therefore, if the temperature is fluctuating inside that dead zone, the heating/cooling systems will not oscillate on and off. A control method investigating the aforementioned notions has been suggested.

Zou et al. [31] gave specific consideration to detecting and classifying the usual behaviours of an inhabitant with time. They evaluated a proprietary algorithmic method called DeepHare that utilised data monitored by cameras linked via Wi-Fi, proximity sensors and accelerometers connected into mobile devices to sense routine human actions. The dataset collected was subsequently treated by means of an extraction network termed the Autoencoder Longterm Recurrent Convolutional Network. The patterns determined from this network comprise both direct data being recorded by the sensors and historical data obtained by decoding several clock series of data as a sole data point, which is next evaluated. In a trial, the algorithm achieved 97% accuracy.

### 3.2. Adaptive Methods

Compared with predictive methods, adaptive methods focus on modifying the energy utilisation of a building based on live feedback.

Lachhab et al. [32] tested the efficacy of various feedback methods based to home ventilation systems. Three approaches of feedback control were considered: ON/OFF, PID and state control arrangement switching. They observed that the state feedback method was the best effective method for warranting a fast-setting time of less than 0.05 s, system stability and reliability. On the other hand, the simple ON/OFF system switching method, which was in fact a variable duty cycle, was unstable and more easily disrupted than the previous alternatives.

Marinakis et al. [28] examined the feasibility for live consumer feedback to control a building’s temperature for the comfort of its inhabitants. Prior to being set to a given temperature, a comfort index identified as Predicted Mean Vote was assessed. This index is a sliding scale with a range of values between −3 and 3, with −3 being cold and +3 being hot, and was established on air and radiant temperatures, relative air velocity, humidity, as well as clothing and metabolic levels of the building’s users. The equations utilised to estimate this index were, in addition, adjusted in real-time so as to provide an index named as Observed Mean Vote. Building occupants are able to utilise a web app to update their personal evaluations of air environments, which is then used to calculate the Actual Mean Vote. The outcomes of a trial scenario disclosed, for example, that the computed comfort level was generally worse than the inhabitants’ feeling of the environments, suggesting that certain extent of scaling is needed with the temperature proposed by the algorithm and the fixed set point.

**Figure 4:**
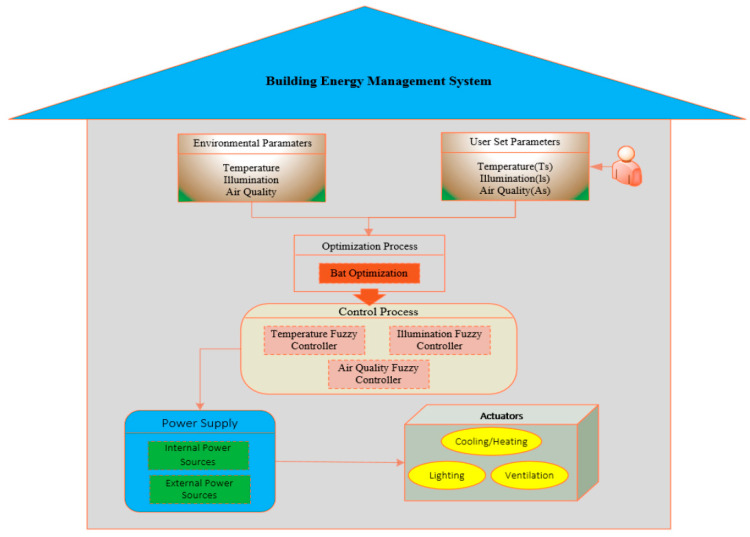
Building energy management system using fuzzy logic approach [33].

Fayaz and Kim [33] have analysed the approaches through which adaptive methods could be utilised jointly with predictive methods that were reviewed earlier in this section. An optimised prognostic model was utilised to assess energy consumption a function of time for different temperature control, air quality and indoor natural illumination parameters. This predictive model was then implemented with a fuzzy controller, which operated in relation to the degrees of truth, e.g., cold, hot, etc., instead of discrete Boolean logic. By computing the error among expected values and actual values for all these parameters, and inputting these error computations into the fuzzy controller, a more correct quantity of power utilisation for these devices can be termed. Figure 4 presents how such a system can be implemented.

## 4. Architecture and Networking

The past two decades have seen the establishment of numerous networking architectures that authenticate security and ensure accurate communication between devices and controllers. Specifics on communication procedures applied in IoT (for example, Bluetooth, Long-Range long-power Wide Area Network (LoRaWAN), Wi-Fi, ZigBee or Z-Wave) applied in IoT can be found in [12,17,30]. The good news is that many of these networking architectures and protocols remain relevant today. A few research examples that confirm the applicability of existing protocols are presented below.

Serra et al. [34] assessed the utilisation of wireless sensors operating on a 2.4 GHz network on a residential IoT architecture to optimise HVAC controls within a home. Every node in the wireless sensor network (WSN) was interconnected through the server, transferring data directly to the IoT application server, rather than over a local modem. The HVAC modules controlled by actuators were then wired to the associated sensors. The complete architecture consisted of (i) a variety of HVAC units; (ii) actuators, which control the HVAC units; (iii) a WSN that transmits measured values of temperature and energy consumption to a gateway; (iv) a gateway (GW) that integrates the energy scheduling approaches and links the on-site network to the Internet, (Namely, it comprises a web server and a database to collect data received at the GW from the WSN or the internet), and (v) An embedded IP machine (e.g., smartphone or tablet) to allow users to interface with the HVAC energy scheduler. The operability and flow of information for optimising temperatures and energy consumption is described by means of the following. The temperature is measured at different positions using the WSN. These quantities are regularly transmitted to the gateway, where the energy scheduling algorithm is applied. This later chooses the arrangement of the functioning HVAC units that minimises the energy cost for specified comfort restrictions and energy price throughout a specific duration. These results are directed over shell commands, to programmable surge protectors (actuators) that trigger the HVAC units, thereby adjusting the actual room temperatures. As mentioned earlier, the gateway hosts a database to store temperature and energy utilisation measurements. Finally, these measurements can be retrieved and viewed by an authorised used remotely on the Internet, using the gateway’s web server to handle the data and communications processes within the remote consumer and the on-site database. Users may also be permitted to interact with the energy scheduler application, adjusting room temperatures for their comfort and convenience though within the pre-decided upper and lower temperature bounds. The connections between the most relevant units were presented in their work.

**Figure 5:**
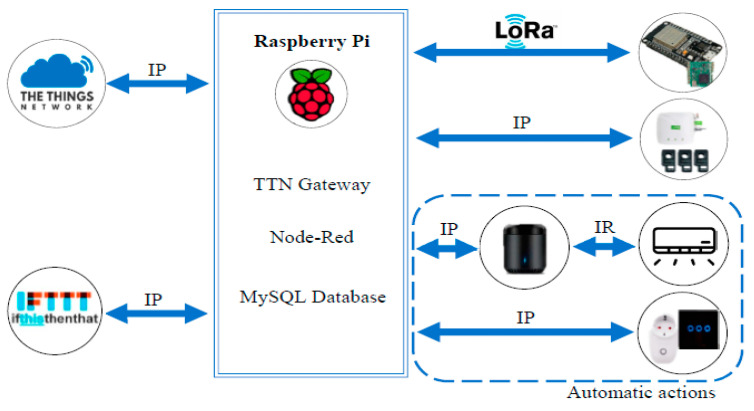
System architecture based on The Things Network [30].

Mataloto et al. [30] evaluated The Things Network (TTN), which is an open source, totally open protocol for IoT applications. TTN allowed the connections between simple hardware arrangements (for instance, the ones operating with Arduino or Raspberry Pi as their central controller), and secure and robust software suites. This stable software suite accepted the simplest of microcontrollers to be converted into a reliable gateway, and it collected sensor data directly from the microcontroller and directed the resulting outcomes back to the microcontroller for actuation. The utilisation of a centralised algorithmic service was at the heart of the stability of the IoT system, as basic code was scripted to the microcontroller itself. Communications in this IoT architecture, i.e., between controllers and units was managed through an ordinary Wi-Fi network using TCP/IP, as depicted in Figure 5.

**Figure 6:**
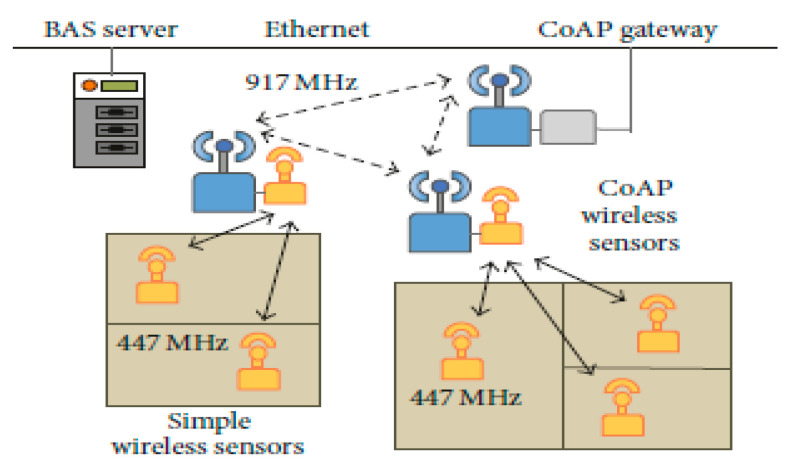
Network using constrained application protocol (CoAP) sensors [35].

Kim et al. [35] analysed a feasible wireless network for house heating. Wireless thermostats and CO_2_ sensors were utilised as substitutes of traditional hardwired sensors. These wireless sensors were then allocated all over the house, covering both indoor and outdoor spaces. Clusters of sensors in close proximity with each other were attributed a restricted application sensor, with which they send and receive data over a 447 MHz band. Bandwidth selection was to decrease total energy use; data going between the individual sensors were not complex, and therefore a lower power bandwidth could be used. All the controlled application sensors were linked with each another and with a central gateway using a 917 MHz band; the higher bandwidth selection to support the additional data that is transferred among sensor clusters. In addition, at the last stage, Ethernet was applied for communications between the central gateway and the house automation server. The house server then utilised the collected data to make inferences and appropriate system-level decisions. Figure 6 shows a visualisation of how the various system components.

Outcomes by Mehta et al. [36] additionally corroborated and confirmed necessity for generic methods and standards for IoT systems to become viable solutions for cyber-physical applications that deal with large amounts of data.

Sodhro et al. [37] explored IoT networks for smart cities that span multiple buildings and vehicles. Their study addressed the issue of resource management when many sensors are communicating simultaneously. They proposed two specific algorithmic structures: Adaptive Bandwidth and Delay-Tolerant Streaming. The Adaptive Bandwidth algorithm was utilised to regulate the frequency whereupon the sensors transferred data in order of reducing power consumption, with the consequence that data transfers required extra time. Delay-Tolerant Streaming while similar in concept, operated in the reverse route: if transmission is extended, power is augmented to increase bandwidth and then reduce transmission time. Additional algorithms may be needed to compute and evaluate the trade-offs between power utilisation and transmission delays in order to manage a sufficiently stable network. The use of these algorithms showed that an additional 37% in power utilisation beyond other equivalent techniques could be saved.

## 5. Case Studies of the IoT Applied to Building Energy Systems

An extensive list of the applications of IoT does not exist. IoT has been shown to be useful in a wide range of applications. Examples include smart cities, buildings, hospitals, homeland security, transportation, education, military, agriculture, industry, manufacturing, shopping and other modern technologies [6]. Each of these applications is a “field of its own”. For example a smart city includes various IoT systems dedicated to parking availability, traffic optimisation, weather preparedness, public transit and building efficiency [29]. This section focuses on one of the most common applications of the IoT, which is to optimise building energy use.

As seen in previous sections, each application requires a custom IoT system in order to be effective. Each IoT system requires sensors that are specific to the environment and data required. Additionally, how the sensors are connected to the controller varies, whether it be short-range wireless (Bluetooth), wired, cellular for long distance or Radio Frequency Identification (RFID) for security as examples. The controller device must be selected such that it has the capabilities required by the application to connect with the sensors and run the software. Next, depending on how much data is to be collected and security concerns it must be determined whether the system will be connected to the cloud for storage and analysis. If the IoT system is in fact connected to external clouds, then, the system requires gateways to the internet. Last, similar to the selection of sensors, any smart devices must be selected that are specific to the objectives of the IoT system in its application.

There are various ways that IoT systems can be applied to improve building energy consumption. This section has grouped the works into the five most common application types of IoT in this area, which are energy consumption monitoring and control, predictive temperature control, occupancy and comfort sensing, controllable devices, and the smart home application. The following subsections go through why IoT should be applied in each way, how it would be implemented, as well as any available results of existing implementations. We conclude with a final Section 5.6 that offers a couple of holistic case examples.

### 5.1. Energy Consumption Monitoring and Control

IoT is an effective tool to monitor and control building energy use. The IoT system can be set up to measure power draw at the source, meaning each piece of equipment can be isolated. Alternatively, the IoT system can look at power demand by different rooms or floors. Collecting this data is useful in a few different ways. First, it will identify which systems or devices are responsible for high-energy usage and may be good candidates to make more efficient. It helps to better understand how certain actions effect energy usage, meaning it could be used to study the results of various energy reduction methods. Second, and potentially most importantly, the application where the IoT system is not only monitoring but actively controlling the power use. This is achieved by allowing the IoT system to disconnect devices from the grid to remove standby power consumption. Third, the IoT system can select when devices can access the power, such as limiting consumption if a task it is not critical. If the option exists, the IoT system can select times of low grid demand to power devices. This would be more cost-effective for the user and help spread community power demand, which allows for more efficient generation.

Pocero et al. [38] described a school building that was selected to install a power monitoring IoT system. Given that there are many school buildings, their solution could be very impactful if rolled out to other schools. Additionally, schools can expose students to energy monitoring methods that could have positive long-term effects. The goal of the system was to produce a real-time platform to collect environmental and energy consumption data from school buildings. The system would also monitor human comfort parameters and the outdoor environment. The system would use a low-cost sensor network distributed throughout the building to more cost effectively monitor. The system connected sensors to nodes (Arduino) that communicate using a wireless radio interface (Arduino XBee) to an IoT gateway device that in turn had a wired connection to the cloud. The power measurement sensors were mounted to the exterior of wires to measure the current passing through them. This current is then used to determine the real power from knowing the voltage and the power factor. See below for an example of current measurement. The whole architecture of the system was built from multiple heterogeneous installations in school buildings. Each installation consisted of a multitude of IoT nodes, which communicated with a cloud infrastructure via an IoT gateway device. Depending on the installation, each schoolroom comprised of one or more IoT node. IoT nodes comprise multiple sensing devices, while the gateway nodes synchronise communications and allow communications with cloud-based services and other Internet linked devices. All devices in their system used a wireless radio interface to interconnect locally and fixed wired interfaces to transfer measurements to the cloud services of the system and receive commands or pattern updates. The overall design configuration for the installation of IoT infrastructure in the school buildings taking part in the project was as follows. (i) Power consumption meter for IoT nodes that are connected to monitor the power consumption of the building as a whole, or within specific floors. (ii) Environmental comfort meter for IoT nodes that are connected in classrooms and other supporting rooms to monitor a set of environmental variables, such as temperature, humidity, activity and noise levels. (iii) A set of IoT gateway nodes that are connected in central locations of the building to bridge the IoT nodes that interconnect using IEEE 802.15.4 with the Internet. To allow exchange between the IoT nodes, they used IEEE 802.15.4 provided by XBee devices linked to each IoT node using the Arduino XBee and XbeeRadio software libraries. All IoT nodes constitutes an ad hoc network and communicate their testing data within the specified IoT gateways.

**Figure 7:**
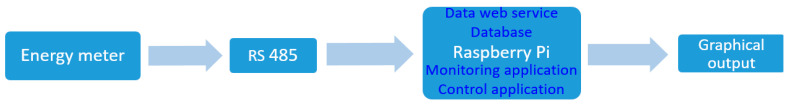
Schematic block diagram of the IoT energy monitoring system.

Mudaliar and Sivakumar [39] presented the application of an energy monitoring IoT system applied in an industrial building setting. A Raspberry Pi was selected to control the monitoring system for its low cost and reliability. The Raspberry Pi was used with Node.js programming language to collect data from current energy meters and save it locally. The data were then accessible from laptop or mobile devices using Grafana. The energy monitoring system facilitated energy conservation measures. The IoT-based energy monitoring system consisted of the existing energy meters, Raspberry Pi, Cloud (database, data web service, control application, and monitoring application) and visualisation systems. Several types of IoT devices such as Arduino, Raspberry Pi, Intel Edison, Mediatek linkit one, NVIDIA Jetson Nano, etc. are accessible to complete the task. For their study, they utilised the Raspberry Pi based system, as they found it to be the best alternative to complete their duty. The energy monitoring system is shown in Figure 7 [39].

Mihailescu et al. [40] explored the development a low cost throughput IoT system for power-driven appliance recognition. The challenge of electric apparatus detection and monitoring is getting greater significance considering the constant increase of house and building automation management systems. Flexible options that challenge this problem could get use in numerous areas, comprising demand-side management, prognostic maintenance, raising of energy utilisation understanding or medical services, where action recognition could be suggested dependent on appliance-level energy use profiles. In their work, the design and formulation specifics of a full scope output for appliance recognition was presented. Different machine learning approaches were compared to best identify appliances with variable consumption patterns. This investigation helps to gain accurate energy consumption monitoring without directly measuring the power use of each appliance.

**Figure 8:**
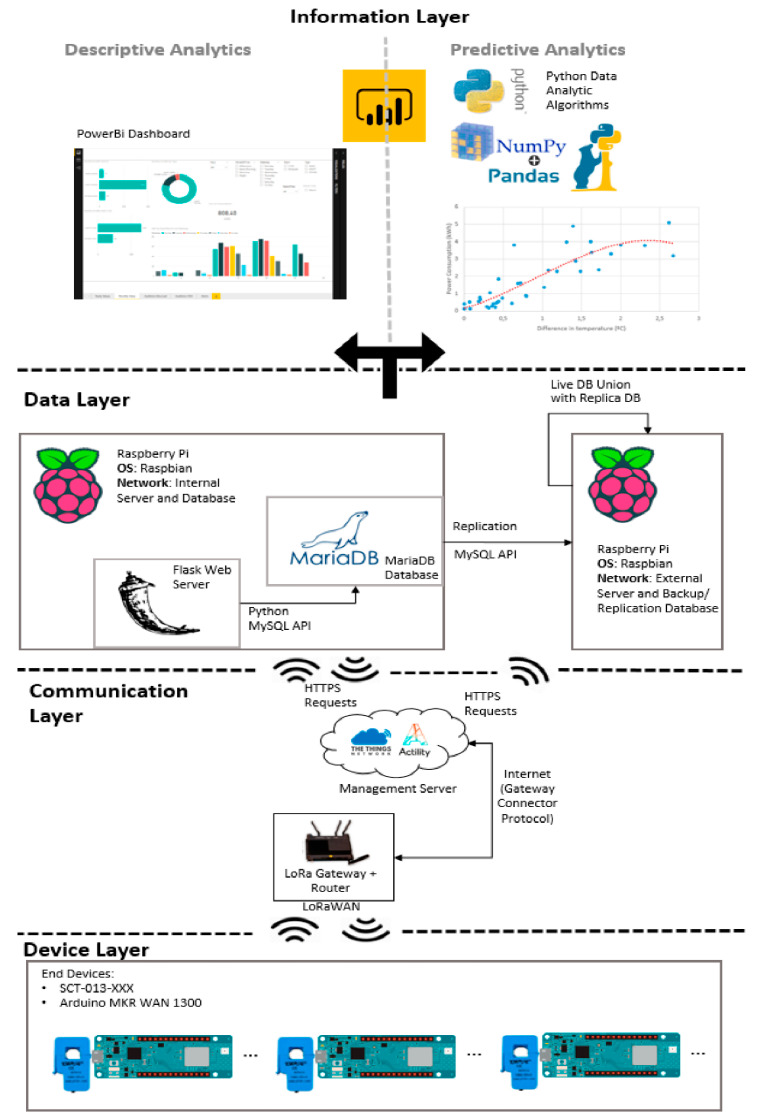
EnerMon long-range (LoRa) IoT system design [41].

Santos and Ferreira [41] described the development and validation of a long-range (LoRa) IoT system named EnerMon used to monitor power consumption. The solution used Arduinos, current transformer sensors, LoRa communication and a Raspberry Pi as an application server. The system can be installed easily at low cost (80€ per sensor) to provide real-time information and analytics to identify energy waste. Due to LoRa's low flowrate transmission, an edge computing method was applied to generate an actual monitoring procedure built on this technology. This method was also endorsed for its simple installation without communication range and complications restrictions, getting it straightforward utilisation in diverse conditions from large complex buildings to smaller users, for instance electric boilers, or just to evaluate the energy consumption of customers in a small tourist residence. The structure is created on standard IoT system’s architecture. It has four key modules presented in Figure 8: (i) Device layer—LoRa (LongRange) end device: The end devices utilise edge computing methods to gather information and next forwards it to the LoRa gateway without requirement for a proximate, small-range transmission network (e.g., Wi-Fi). (ii) Communication layer—LoRa gateway: gets uplinks over end devices and transmits that information to at least one application servers, then, as well, transmitting downlinks to end devices, if there is a requirement to transmit a command. (iii) Data layer—application server and databases: receives information over the gateways, treats this information and saves it in a database while interacting with the end-device through the LoRa gateway. (iv) Information layer—dashboard and analysis: the data saved is employed to produce a console that gives data in the manner of comprehensive charts along suitable filters, with the analysis executed using PowerBi’s control panel in conjunction with Python to better understand the data. Table 4 provides the core variances between each communication protocol usually utilised in IoT works. They advised that every exchange procedure could be utilised on various kinds of cases; there is no superior procedure in general, though, there will always be particular procedures that could be better utilised for given conditions [41].

Through the growth of IoT, further smart gadgets/appliances will be incorporated into residential buildings within smart buildings/cities, which proactively take part in electricity markets through demand response (DR) programmes to cost-effectively control energy so as to encounter this growing energy demand. For instance, Hafeez et al. [42] developed an energy management strategy by means of price-based DR programme for IoT-allowed residential buildings. They proposed a wind-driven bacterial foraging algorithm (WBFA) that is a hybrid of wind-driven optimisation (WDO) and bacterial foraging optimisation (BFO) algorithms. The WBFA algorithm can help to make IoT-enabled buildings and cities more sustainable. The programme managed the power usage of IoT enabled smart appliances to avoid peak demand times, minimising cost and maximising user comfort.

Massano et al. [43] described a study that uses the IoT to detect both the indoor air temperature and electricity utilisation of HVAC systems. These data were used to acquire the thermal properties of buildings and forecast the indoor temperature profile. It was found that the system was able to predict indoor temperature trends for the next 24 h with an accuracy of 2 °C.

**Figure 9:**
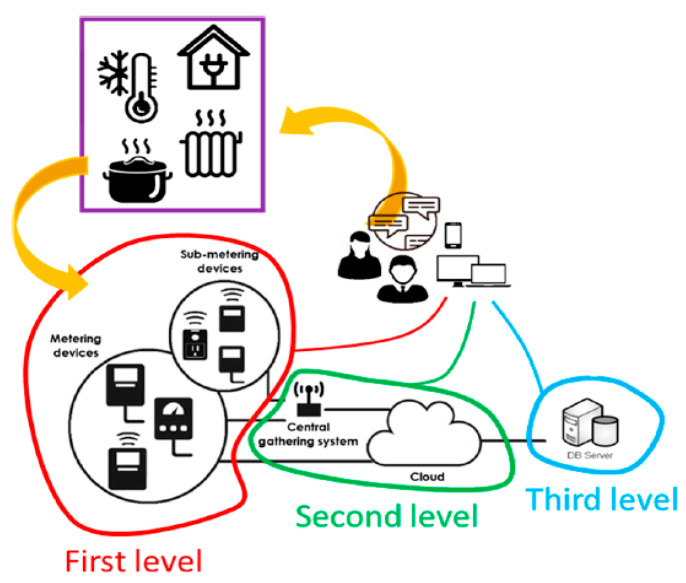
Schematic of the IoT-integrated tool [44].

Dell’Isola et al. [44] investigated the issues of deploying an IoT framework that would gather, process and transmit energy consumption data correlated to the indoor ambient air temperature data. The goal of this framework is to increase awareness of both property owners and tenants to energy consumption though effective billing. As well as to increase awareness, the system could be used to show which landlords maintain suitable living conditions for their tenants. The integrated IoT system created by the authors comprises of three layers. The first layer is characterised by measuring and sub-measuring systems for collecting energy use data from electrical, thermal and natural gas appliances (nodes) of the related facilities. The second layer is the data concentration by wireless personal area networks (ZigBee protocol) and remote transmission data with the building router linked to the Internet. Smart meters could as well promptly interconnect through the cloud. The third layer is the web-based data management supplying parallel outputs for data access, storage, analysis, and processing. Especially, in the third layer, data for customer view are managed through generating reports, along with actual displays by means of console. Therefore, the IoT instrument integrates and saves data, in this manner: (i) The evaluation element saves data from diverse sources (i.e., electric, thermal, and gas energy utilisation and production). (ii) The configuration element saves information from diverse external sources (i.e., energy prices, weather data and consumer’s behaviour). Figure 9 illustrates a basic outline of their IoT combined system.

### 5.2. Predictive Temperature Control

Predictive temperature control is a useful achievement for reducing building energy consumption, and an IoT infrastructure is an excellent way to accomplish this. When the HVAC systems of buildings are controlled based on predictions, they are more efficient than typical reactive HVAC systems. A predictive controller knows information about daily patterns of heating and cooling due to the sun, weather, occupancy, operational devices and various other factors. As an example, the HVAC system can know to not heat the building until immediately before occupancy or know to let the building cool before a heat releasing process. The IoT system is also be used to better detect the performance of the HVAC system in specific locations.

Luo et al. [45] surveyed the many platforms available in the literature for energy management system in smart buildings [46,47,48,49,50] and presented the development of an IoT-based platform that would produce day-ahead prediction of building energy demands. The predictive model would be based on the hybrid of k-means clustering and an artificial neural network. To facilitate the collection, processing, analyses and presentation of IoT measurement data, the suggested IoT-based big data platform comprised of four distinct layers: a sensorisation layer, a storage layer, an analytics support layer and a service layer. The layout of their four-layer IoT-based big data platform is provided in their paper. That structure could as well gain the high-performance computing in exchanging through huge sensors throughout an extended time. The IoT system would provide temperature information for walls, windows, grounds, roofs and indoor air. The extensive IoT sensing improves the accuracy of predictions and helps to make the building’s HVAC more efficient.

**Figure 10:**
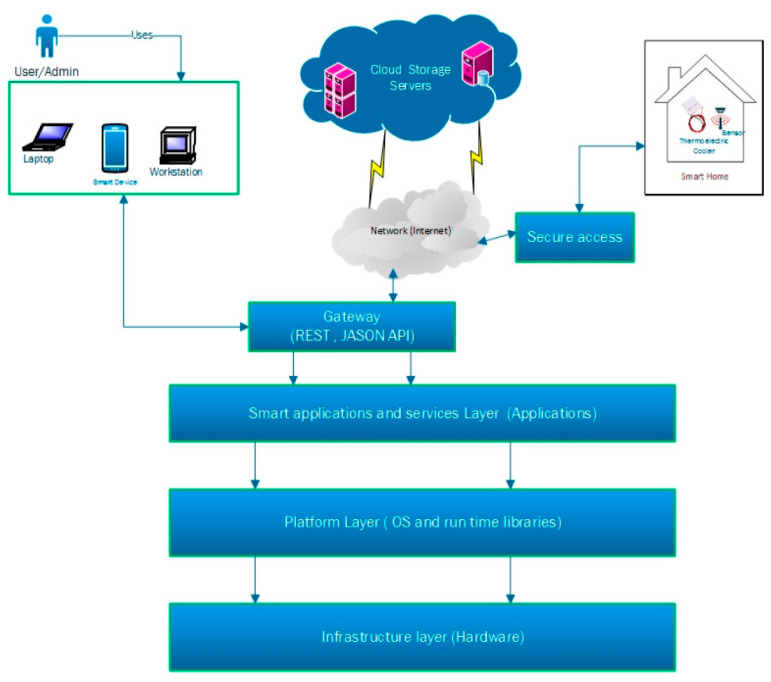
Developed thermoelectric air management architecture: integrating IoT and cloud computing [51].

**Figure 11:**
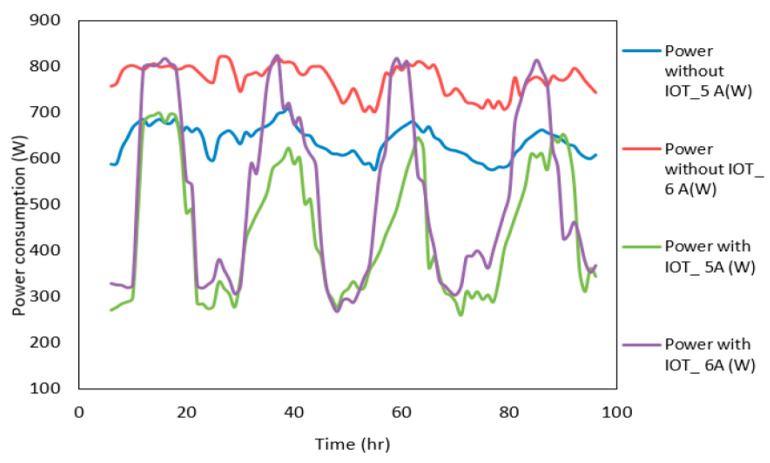
Power consumption with and without IoT-based thermoelectric air conditioning system for two input power operations [51].

Irshad et al. [51] investigated the performance of a thermoelectric air conditioning system that is controlled by an IoT system. The IoT system consisted of an Arduino microcontroller using temperature sensors connected with RF modules and controlled the indoor climate based on the outdoor climate. The system used the IoT platform, which was based on three key principal modules: things (sensors), the gateway and the cloud (networks). The complete system flow, i.e., IoT sensor position, central device position and data flow, is illustrated in Figure 10. The IoT sensor was situated in the lab, which was securely linked to the cloud server. Sensor data are deposited on the cloud server and later analysed via the in-house building analytical application. To safeguard data transfer among various networks, they employed the Rivest, Shamir, Adleman (RSA) encryption procedure, which is a powerful asymmetric cryptography algorithm for encrypting/decrypting data. The system was found to improve cooling capacity by 14%, coefficient of performance by 46.3%, and allow for better microclimate control within the building (Figure 11).

Zhao et al. [52] proposed creating a thermal comfort control system for buildings that is centred on the IoT and artificial intelligence (AI) technique. Various algorithms and prediction models were presented for the control system. The system can automatically carry out a sequence of actions over IoT hardware devices, which were situated at multiple positions in the building with main units. The code was built using Python to create a model for energy consumption forecast with ambient factors such as temperature, humidity, radiant temperature and air velocity on thermal comfort values. Using simulations, it was found that the performance of the IoT system was superior to traditional control in terms of both energy saving and comfort.

Lee and Yeo [53] provided a solution to condensation occurrence in buildings using an IoT real-time sensor control solution. Moisture in the structure of buildings causes damage if it is not removed. The system was designed to sense moisture and react by controlling doors and ventilation to the area. The study provided a solution for reactive condensation control in vulnerable spaces.

**Figure 12:**
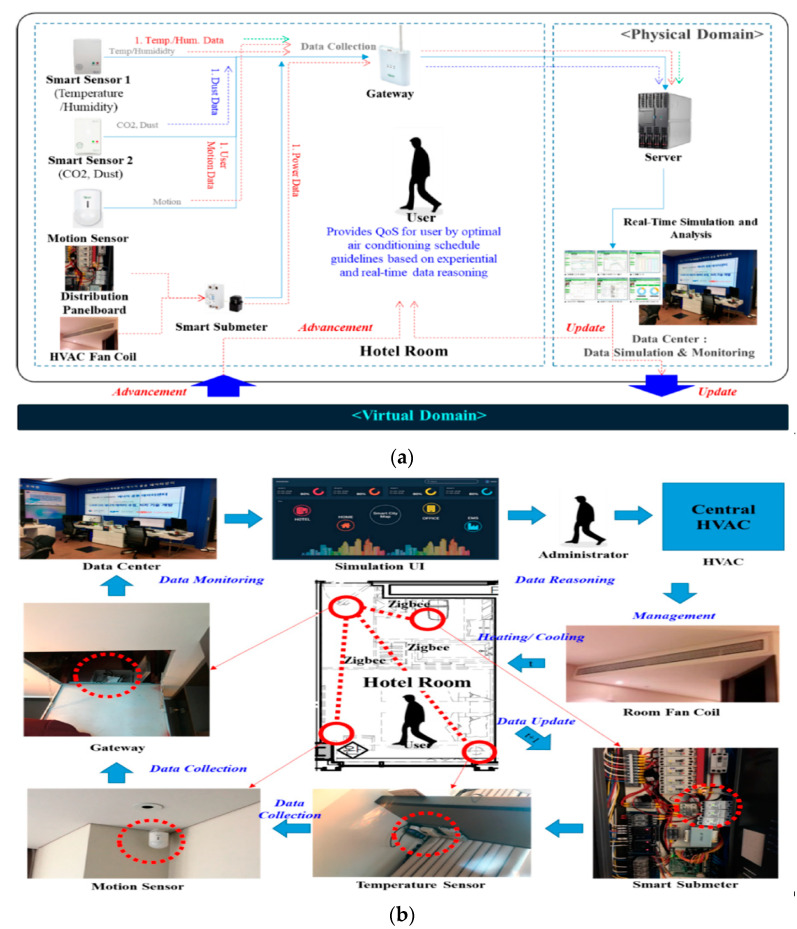
Structure of the IoT system: (**a**) system configuration and (**b**) system flow and installation [54].

**Figure 13:**
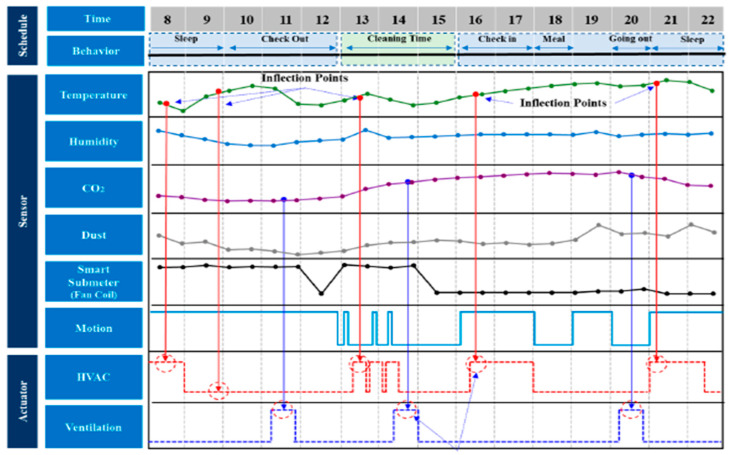
Sensor readings and actuator responses of IoT system [54].

Park et al. [54] described an IoT data collection system used in combination with reinforcement learning to improve energy use in a building. A test case of a hotel was presented where the IoT system collects, analyses and infers energy usage data to create an HVAC scheduling method. The AI system can analyse energy usage data, predict future energy requirements and establish an appropriate energy saving policy. Figure 12 shows the arrangement of the IoT system that was mounted in the hotel environment. All IoT sensors utilised communication based on Zigbee, which is an IEEE 802.15.4 standard-based wireless network technology. Real data transmission speed was measured and confirmed to be over 2.5 kbps (ACK transmitted _ ACK received). Figure 13 depicts the readings of various sensors that the system collects and uses to make decisions.

**Figure 14:**
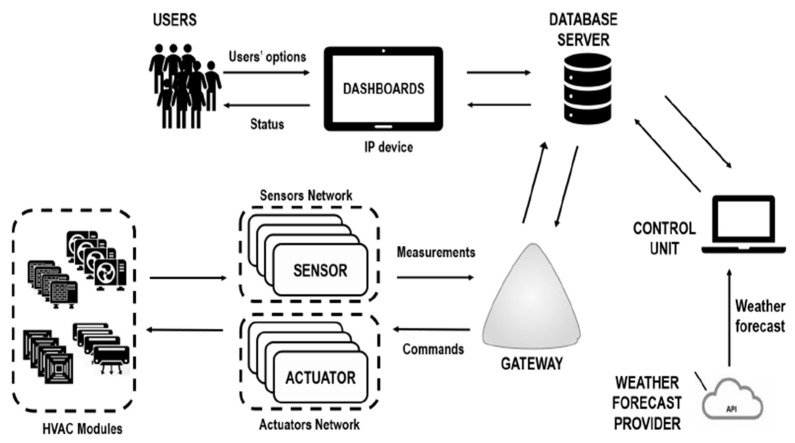
Schematic of system arrangement of IoT architecture [55].

Carli et al. [55] proposed an IoT-based architecture for the application of model predictive control (MPC) of HVAC systems in actual situations. The examined MPC algorithm was optimised online, in a closed-loop control mode, both the indoor thermal comfort and the associated energy utilisation within a single zone situation. Over their IoT system, the sensing, control and actuating subsystems were all linked to the Internet, and a remote interface with the HVAC control system was made available to users. Their sensors and actuators interconnect with a remote database server and a control unit, which supplies the control actions to be actuated in the HVAC system. Users are able to decide remotely the control mode and related control levels of the system, while comfort and environmental indicators were transmitted via the Internet and presented on the users’ interface. The IoT-based control architecture is realised and tested in a campus building at the Polytechnic of Bari (Italy) in a proof of concept purpose. The efficiency of the control algorithm was evaluated in the real setting appraising both the thermal comfort outcomes and the energy savings with reference to a conventional thermostat control mode. Figure 14 presents the system diagram of the IoT architecture.

**Figure 15:**
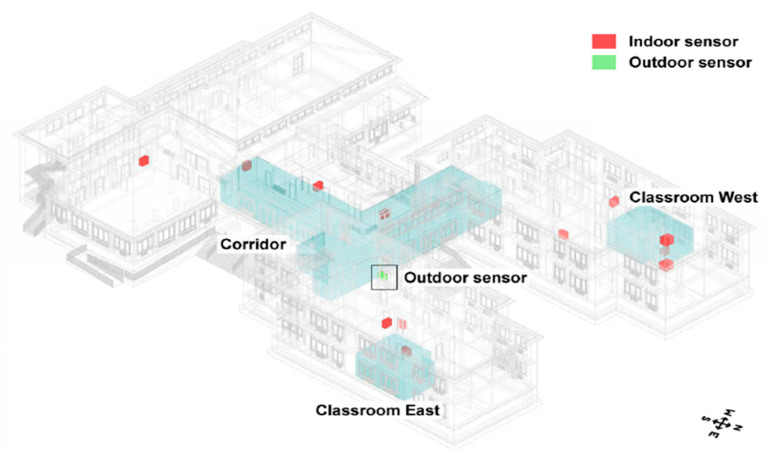
Building sensor layout of case study [56].

Aliberti et al. [56] assessed an IoT-based solution for indoor air temperature forecasting. Forecast-based technologies, for instance, demand response and demand side management, are used to reduce energy waste. The solution uses an advanced nonlinear autoregressive neural network for making predictions based on IoT collected data. The predictions were found to be both accurate and robust. Figure 15 illustrates the layout of sensors within the case study building.

### 5.3. Occupancy and Comfort Sensing

There are various ways that user comfort can be detected using IoT systems and otherwise. User comfort is important to measure as this must be maintained or improved during the implementation of an energy consumption reducing IoT application. The addition of occupancy and comfort sensing allow the system to perform to exactly what is desired by users, instead of over heating, cooling, lighting, etc. Additionally, occupancy and activity details can be used to tailor energy systems only to regions that require it in either the long- or the short-term. This could be as simple as automatically turning on and off lights or allowing predictive HVAC control based on long term occupancy patterns.

As previously seen, Serra et al. [34] addressed the efficient energy consumption management of HVAC systems in smart grids with variable energy pricing. They proposed an energy scheduling scheme that minimises the energy consumption cost for a specific time interval, considering the energy price, and a group of comfort constraints, namely, a range of temperatures based on user’s choices for a particular room. The HVAC system considered user preferences for specific rooms and specific temperatures to maximise comfort. Besides, the HVAC system controls can be monitored and adjusted remotely for “on the fly” modifications to keep the HVAC system efficient. The algorithms have been realised in a real testbed, underlining the prospective improvements that can be reached based on both energy and charge.

Marinakis et al. [28] presented a temperature setpoint decision support system for building energy manager based on occupant feedback. The system used a Thermal Comfort Validator web app that predicted the mean votes comfort theory based on real-time occupant feedback. The system is poised to achieve energy savings based on data collection and processing while guaranteeing high levels of comfort.

Tomat et al. [57] compared three solutions to providing occupant comfort and energy efficiency to buildings. The first a basic IoT hardware reactive sensing solution, next a simulation-based modelling and last a crowdsensing study. The crowdsensing solution allows users to express their sensations using IoT devices providing the system with comfort data. Results disclosed that crowdsensing has an encouraging prospect in the exploration through the IoT, even if several technical steps forward are required to attain a suitable application to the thermal comfort issue.

Vanus et al. [58] investigated how an IoT system can passively sense building occupancy through the monitoring of CO_2_, temperature and relative humidity measured using standard functioning measurements through the KNX (Konnex, Standard EN 50090, ISO/IEC 14543) technology. They described the structure and development of a software means for certifying connectivity of the KNX technology and the IoT IBM Watson platform in real-time for storage and visualisation of the determined quantities by means of a Message Queuing Telemetry Transport (MQTT) protocol and data storage into a CouchDB sort database. Within the frame of the inhabitation establishment approach, the forecast of the CO_2_ concentration profile from the measured temperature and relative humidity quantities, were executed by means of numerical techniques of linear regression, neural networks and random tree (using IBM SPSS Modeler) with a precision greater than 90%. To further the accuracy of the prediction, the use of elimination of additive noise from the CO_2_ signal forecasted by CO_2_ using the least mean squares algorithm in adaptive filtering technique was utilised in the recommended method. The detection was found to have an accuracy higher than 90% from approaches of linear regression, neural networks, and random tree. By recognising occupancy and activities, the intelligent building can be automated jointly with energy savings.

### 5.4. Controllable Devices

Controllable devices represent a wide range of applications within building efficiency. An IoT system at minimum contains sensors that sense and transmit data about their environments while controllable devices allow for an automatic response to the data. Devices relating to building energy primarily include HVAC, lighting, fans, doors/windows, appliances and actuators that can perform limitless functions such as shades. Intelligently controlling these devices can lead to significant energy savings and allow an IoT system to be responsive to the data it is collecting.

King and Perry [22] provided a building energy IoT overview as well as experimental results and recommendations from different IoT systems. The article listed and described the IoT devices that it has considered: HVAC, lighting, plug loads and window shading. As an example, it was found that advanced lighting controls resulted in 45% energy savings and a web-based lighting management system resulted in an additional 20–30% savings.

**Figure 16:**
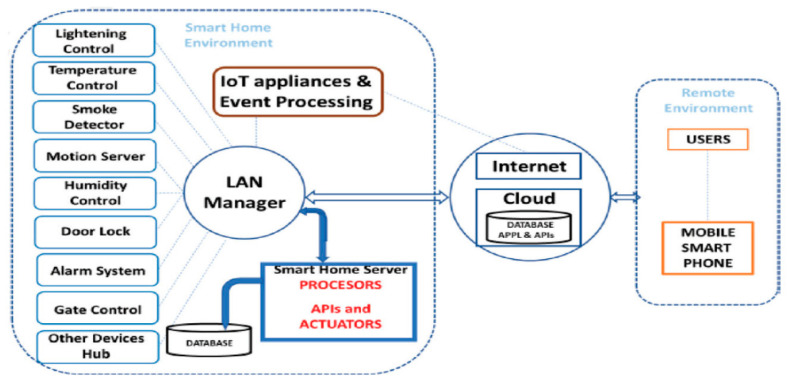
Schematic of smart home incorporating IoT and cloud computing [59].

Domb [59] discussed IoT system control using cloud computing and intelligence as opposed to control from smartphone or web applications. They presented the various home devices that can be controlled by actuators such as lights, fans, appliances and home security. They detailed a configuration of three components to build a robust method of an advanced smart home concept and implementation. Figure 16 illustrates the smart-home core modules and their interconnectivity. On the left block, in the smart home setting, is presented the typical devices connected to a local area network (LAN). This layer allows the communication between the components and outside of it. Connected to the LAN is a server and its database. The server controls the components, logs its activities, delivers reports, responds queries and performs the applicable commands. For further inclusive or regular tasks, the smart home server transmits data to the cloud and remotely triggers tasks in it using APIs, application programming interface procedures. Besides, IoT home appliances are connected to the internet and to the LAN, and therefore extending smart homes to include IoT. The connection to the internet allows the consumer or resident to connect with the smart home to acquire up-to-date information and remotely actuate tasks.

Mahbub et al. [5] discussed a typical IoT application of an intelligent autonomous lighting and air ventilation system. The system was capable of monitoring temperature, humidity, CO_2_ concentration and from this can control an energy-aware self-sufficient lighting and ventilation system. The system with HTTP protocol logged data to cloud servers and can be monitored over smartphones or PCs. Additionally, the system was able of logging real-time data into the cloud server over which a consumer can as well display, in real-time, from any location in the world. As it was a wireless gadget, interaction with the control system occurred through the GSM (Global System for Mobile Communications) and Wi-Fi network, using the modern HTTP protocol. A study performed on energy consumption and economic analyses of the suggested system showed its effectiveness.

**Figure 17:**
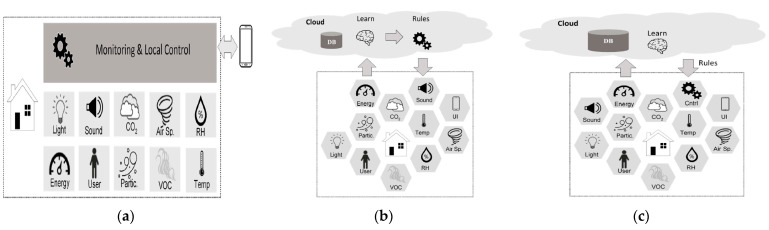
Comparison between various computational architectures for building automation: (**a**) conventional method, (**b**) full cloud computing method and (**c**) edge computing method. Air Sp.: Air speed; DB: database; RH: relative humidity; UI: user interface; VOC: volatile organic compound [60].

Rinaldi et al. [60] proposed the concept of installing a smart building IoT system during the construction phase. The system was capable of sensing and reacting to stimulus. As an indication of the system capabilities, an intelligent window was developed that monitors indoor and outdoor conditions and reacts with motorised opening and shading. The window defined the best rules for opening and shading based on users’ preferences and efficiency targets. The main differences between the solutions depended on where the “intelligence” and the data were situated. Figure 17 illustrates the three diverse evaluation architectures. The first one (Figure 17a) is a categorised method succeeded by conventional home automation system vendors. A neighbouring controller is in charge for the data acquisition from the sensors. According to the arrangement of the consumer, the controller refers the rules to actuators. The consumer is able to interact with the automation system over a user interface (UI) that is usually an application. In this configuration, the data and the “intelligence” are situated in the customer’s house/edifice. However, the rational method necessitates the combination of data based on diverse sources to understand the customers’ behaviours. In addition, the on-site controller usually does not possess the data processing capacity needed to apply the majority of cutting-edge AI algorithms. The second configuration (Figure 17b) utilise cloud-computing technique for building automation. This method is the favoured path pursued by the major hi-tech enterprises, like HomeKit from Apple, Google Home by Google and Home Assistant from Amazon (refer to Appendix A for references). Lately, an effort to state shared application programme interfaces (APIs) for the automation of building energy systems was recommended by the project Connected Home over Internet Protocol (IP) [61], sponsored by big hi-tech enterprises and Zigbee Alliance. In this architecture, all the data produced by the sensors are transferred to remote cloud servers, where the data are deposited in no-structured query language (NOSQL) databases [62] and utilised by cognitive algorithms to gather information regarding consumers’ behaviours that is then further utilised to enhance the comfort and the energy reductions of the monitored building. In this scheme (Figure 17c), part of the intelligence is situated nearby in the building. The nearby controller is in charge for applying the procedures implied by the remote cognitive intelligence [60].

### 5.5. Smart Home Application

Residential buildings make up a considerable proportion of energy consumption in the building sector so taking steps to improve efficiency are valuable. Additionally, homeowners desire their homes to operate in a smart and efficient manner for environmental and financial reasons. There are various IoT technologies that can be installed by homeowners to achieve this goal. In many cases, it is the same IoT technology used in commercial buildings, but with additional requirements such as security, robustness, ease of use and control methods. The IoT solutions allow the home to be more efficient, comfortable and consume power when it is economical to do so.

**Figure 18:**
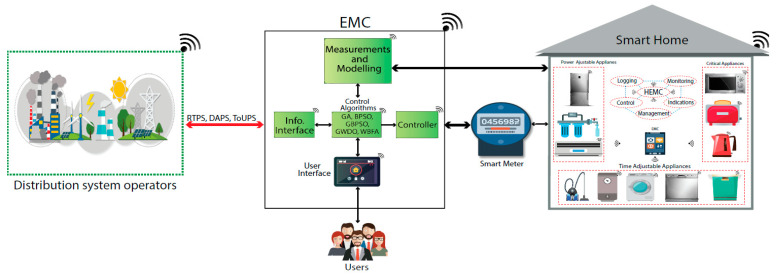
Energy management framework for residential buildings [42].

As previously mentioned, Hafeez et al. [42] presented a demand response programme for residential buildings with IoT systems. Their programme managed the power usage of IoT enabled smart appliances to avoid peak demand times, minimising cost and maximising user comfort for the homeowner. The programme is a wind-driven bacterial foraging algorithm that can help to make IoT enabled buildings and cities more sustainable. The schematic diagram of the proposed framework showing the connectivity between the smart home, user and power grid is illustrated in Figure 18.

As seen previously, Domb [59] discussed an IoT system control using cloud computing and intelligence as opposed to controls from smartphone or web applications. They presented the various home devices that can be controlled by actuators such as lights, fans, appliances and home security. A general overview of how to apply IoT to residential homes is also provided.

Chilipirea et al. [63] identified the potential benefits of applying IoT to residential homes, but acknowledgeds that system robustness is a major obstacle since each sensor has different capabilities and functions. They proposed a model to switch faulty devices with other not-directly compatible ones. As an example, a faulty security camera could be replaced with a door opening sensor and a heat sensor.

Filho et al. [64] reported an investigation of smart home IoT systems that integrate Wireless Sensor and Actuator Networks (WSANs) and computational intelligence methods with an emphasis on energy efficiency. They identified and discussed other studies on the topic, including challenges and providing solutions. They concluded that solutions based on sensors and actuators with a computational intelligence had arisen as a smart method to improve building energy efficiency. The typical architecture of a WSAN associated with computational intelligence in a residence, in which sensor nodes are distributed everywhere in the building to gather data, is presented. In this setting, there are numerous sensor nodes and wireless actuators positioned in a house to collect information from the occupant, which could include the usage of a smartphone to acquire data of the user’s behavioural conditions. From the data collected from the node and/or smartphone, a set of methods derived from statistical models are utilised to learn and actuate in the house. As result, the WSAN has an architecture that presents many tasks, which are being extensively examined by the academic community [64,65,66,67,68,69].

Mocrii et al. [70] provided a general description of an IoT-based smart home through a review of major technologies. They have focused sections within their paper on software, communication, components and security technologies in IoT based smart homes. An additional section provided the current challenges and solutions on the topic.

### 5.6. Examples of Case Studies of the IoT Applied to Building Energy

In addition to previously literature, two case studies of the IoT applied to building energy systems are provided.

**Figure 19:**
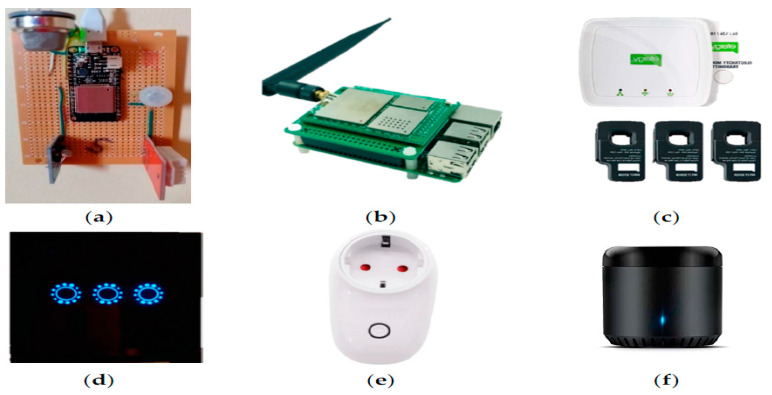
Measurement and control hardware: (**a**) sensor board, (**b**) Raspberry Pi with LoRa Hat, (**c**) energy monitoring system and amperometric clamps, (**d**) smart wall switch, (**e**) smart power socket and (**f**) Wi-Fi/Infrared [30].

**Figure 20:**
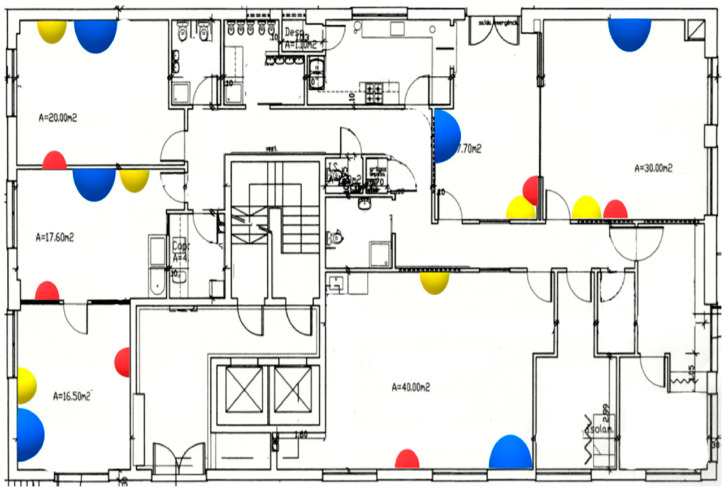
Kindergarten layout with sensors shown in yellow, air conditioning units shown in blue, and infrared emitters shown in red [30].

As previously seen, Mataloto et al. [30] presented a case study application of an energy management IoT system verified in a kindergarten school for the duration of 3 years. An open accessible data set was produced from the sensors that collected data, for instance, temperature, humidity, luminosity, air quality and motion. The sensors were battery operated with long-range communication interfaces. The system collected sensor data, visualised it and automatically controlled the buildings energy systems. The outcome of this experiment was 20% energy savings. A building layout is shown below with the sensor locations. They presented a study on optimising energy utilisation by installing an energy management system employing the current IoT platform through a layered architecture. The proposed platform, named LoBEMS (LoRa Building and Energy Management System), was assembled with the objective of showing a joint platform that will incorporate various supplier secured systems composed by custom sensor gadgets, supplying crucial data for the purpose to enhance complete building effectiveness. This method was verified in a kindergarten school for a period of three years. The sensors, which supply ambient data to the energy management system were comprised by battery-driven sensors coupled to a system on chip with a LoRa transmission interface. These sensors collected ambient data like air quality, luminosity, humidity, motion and temperature. An energy monitoring system was also included. This adaptable method could simply be implemented in buildings, using present setups, without necessitating any remote automation capacities. The appraisal of the platform enabled a 20% energy reduction according to the integrated energy reduction procedures. Figure 19 presents the measurement and control hardware, and Figure 20 depicts building layout with sensors [30].

**Figure 21:**
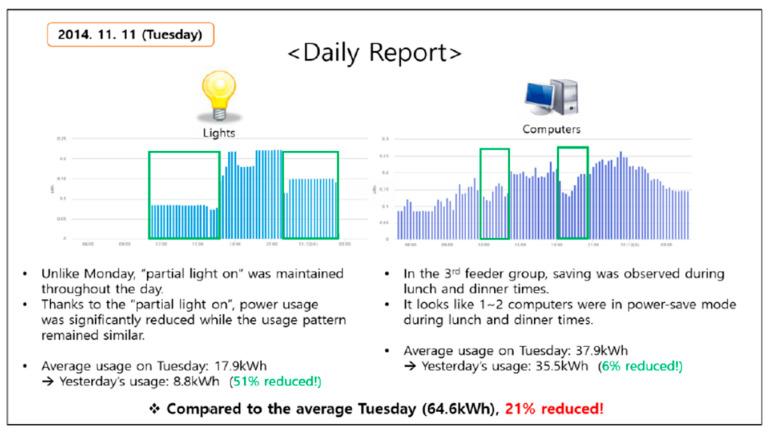
An example of the daily report delivered from IoT system analysis [71].

Han et al. [71] presented the findings of an energy saving experiment in a university building that consisted of a power consumption monitoring IoT system. It was found that the quantity and resolution of power consumption sensing was critical to result in behaviour change related energy savings. The study identified ways to analyse behavioural changes as seen through the uncontrollable factors in a university building. It was found that during system operation energy savings were 25.4% and after the study was completed 10% energy savings were recorded for 44 days. Figure 21 below illustrates an example of a daily report that could be made from the IoT system data.

## 6. Security

IoT systems are challenged by a varied group of security threats that could alter their operations and data protection in domestic uses cases. Therefore, further security indicators, for instance, confidentiality of data and robust functionality in case of a possible risk of being hacked, safety and private security, need to be entirely addressed towards resolutions.

In the work done by Brun et al. [72], the rejection of sleep attack was investigated. Rejection of sleep attacks malevolently retain sensors and controllers when they would otherwise be resting. Given that the majority of IoT systems are quite restricted about coping with power use and speed, this might substantially decrease the lifetime of the components and sensors in the setup. The methods examined to properly detect and recognise an occurrence are reviewed in their study.

With the intent to prevent attacks from occurring, it is necessary to build powerful control and verification procedures across the system. A number of feasible verification procedures were delineated by Andaloussi et al. [73]. One was the centralised method wherein entire verifications were completed at the central level, i.e., at the controller, with no resource constraints, though with a single spot of failure. The alternative approach is the hybrid method wherein authentication occurred over both the sensor level and controller levels, but had numerous points of breach, therefore increasing the risk of safety failures.

Alkhalil and Ramadan [74] appraised a wide variety of possible security attacks. Though wherever the typical owner is tolerable of data breaches that disclose their house heating demands, information may be inappropriately retrieved and utilised to understand further characteristics of their family living-information that they might be unwilling to reveal widely. Another problem is the rejection of service, in which the IoT system specially loses connection through the control system, which is required to manage energy consumption. Conventional encrypting techniques utilised through common electronic machines could still be applied in an IoT environment, but the central processing units tend to be significantly constrained than other gadgets, resulting in significant delays. It is essential to reach a trade-off between computing time and the required degree of safety necessary under the architecture for every usage—as there does not exist any commonly best solution for all IoT infrastructures.

Han et al. [75] evaluated a method of preserving security in the controller by exploiting Blockchain. The distinctive and innovative characteristics and capabilities of Blockchains allow the amount of a bigger stage of encryption and time stamping in the controller, thus making it more difficult to fabricate or alter information maliciously. This method is judiciously harmonious with the centralised approach considered above, as it strengthens the specific target of blockage without the concern of resource constrains. Using this procedure, process is improved, authorising for up to 10,000 trades per second.

Dasgupta et al. [76] focused on the consumer privacy aspect of smart home IoT systems. Their paper goes through what are the major privacy concerns as well as where privacy solutions currently exist. The findings of the study are to help users and developers deal with privacy concerns of their IoT systems.

Hasan et al. [77] compared the performance of several machine learning models to predict security concerns in IoT systems. The machine learning models that were compared are Logistic Regression, Support Vector Machine, Decision Tree, Random Forest and Artificial Neural Networks. Attacks types that could be detected include Denial of Service, Data Type Probing, Malicious Control, Malicious Operation, Scan and Spying. The system achieved a 99.4% test accuracy for three of the machine learning models.

Casola et al. [78] described how solving security concerns is critical to the success of IoT. They proposed a solution in the form of a monitoring tool for IoT systems based on the Montimage network. The solution was validated within the H2020 ANASTACIA project to show IoT monitoring capabilities.

HaddadPajouh et al. [79] presented a detailed survey on IoT security issues, limitations, requirements and solutions. Their study builds upon a taxonomy that taps into the three-layer IoT architecture as a reference to identify security properties and requirements for each layer. The study classified potential security threats from an architectural view and provided solutions based on this grouping to assist in the creation of IoT systems, specifically on how to address and adopt best practices to avoid the current IoT security threats on each layer.

Among the infrastructural IoT methods, Radio Frequency IDentification (RFID) has been utilised to allow the proliferation of and communications within IoT networks. Nonetheless, the RFID methods generally experience security problems owing to the intrinsic flaws of underlying wireless radio communications. One of the foremost security concerns is the authentication vulnerability from jamming attacks. To challenge the vulnerabilities of key updating algorithms, Mbarek and Pitner [80] advised an effective authentication method based on the self-adaptive and mutual key updating. They assessed the performance and applicability of the solution with a systematic simulation by considering the energy consumption, authentication failure rates and authentication deferrals. The feasibility and relevance of this solution were verified by applying the proposed authentication method in smart home IoT systems.

A smart home is characterised by the occurrence of a huge number of small, low-power devices, in addition to more standard devices. Based on the IoT pattern, all of them are anticipated to be constantly connected to the Internet in order to supply improved services. In this situation, an attacker can damage both the network security and the customer’s security/privacy. Conventional security processes are not appropriate, as they are too arduous to install and are either too weak to efficiently safeguard the consumer or too restrictive for the new services efficacy.

**Figure 22:**
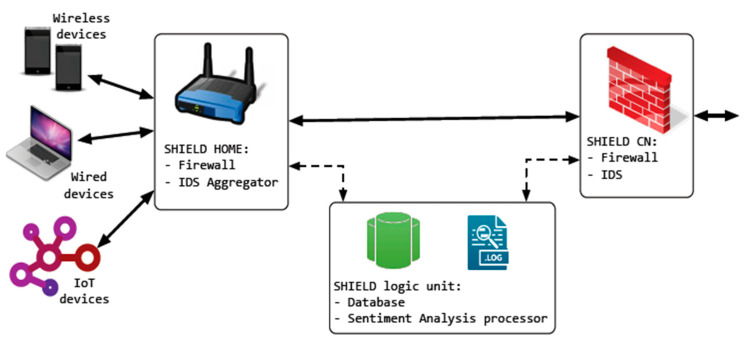
SHIELD IoT security system architecture [81].

Pecorella et al. [81] presented a security system that adapted to the perceived security risk. It is stated that one size fits all security solutions are either too weak to be effective or too limiting of the normal IoT operation. The security risk is determined based on the internet service provider and the user’s cooperation. The security system is based on firewalls and detection systems on both the IoT system and the internet service provider. Figure 22 shows the security system architecture named SHIELD.

## 7. IoT Limitations and Challenges

IoT solutions generally have inherent limitations and challenges, solutions and work arounds exist in many cases. Common issues can present from having a sensor and device network of a very diverse nature as each perform different functions. This is problematic due to the wide variety of issues that could present. Additionally, IoT systems must communicate to each other and wired solutions are not always possible. Connection issues due to interfering structures or unstable internet are common. Another limitation of IoT systems is that they can produce extremely large data sets that require management and storage to be effective. Storage of data is finite and expensive [82].

Other challenges of IoT systems are presented in the previous section, with the largest challenge to IoT systems being security, as well as limitations specific to building energy applications.

There are challenges faced by IoT systems specific to the building energy application. A major challenge in itself is the adoption of smart homes by customers. It was found that compatibility, perceived ease of use and perceived usefulness were the factors that influence purchasing decisions. It was also found that younger customers were less likely to purchase smart homes. The main reasons for a lack of adoption were technical and social indiscipline, requirement for adjustment and familiarisation from homeowners, lack of and limited assistance for learning to practice and a deficiency of indication of significant energy reductions and a probability of energy amplification [74].

Challenges and limitations are specific to each building energy application. In the case of power consumption monitoring one problem is any sensor downtime can have significant errors in the data sets, so this must be monitored. In the event of sensor malfunction, a replacement must be available and easy to install. This also requires that each sensor is customisable to the different power monitoring applications [41].

A crucial interrogation, which challenges this review paper, is its relevance. Do residential or commercial consumers even require understanding these diverse technologies that comprise an IoT system for energy management? This point turns out to be yet further critical in the view of “end-to-end” IoT energy management systems from technology powerhouses, for instance, Google and Amazon or others (Appendix A). This question is addressed as follows: As context, it is a precise characterisation that many of these single-vendor, “closed-loop” services are interesting in various forms: they are competitively priced, simple to install, assisted by certified partner ecosystems, supported by advanced AI/ML control algorithms, and above all have “user-friendly” interfaces. Nevertheless, there are numerous disadvantages that these single-vendors, registered systems present. They “lock-in” end users and pose important hindrances and known, and fewer anticipated costs, which restrain the capacity to substitute vendors. Several of these services restrict functional controls so as to fit their scalable designs. This may be less of a concern for residential customers, but are possibly more crucial, particularly for greater commercial facility operators. More significantly, these IT vendors at this point, possess client data, a direct weakness of which is that they might just preview analytics that might be self-serving to their business benefits. In consideration of these important weaknesses of proprietary “closed-loop” systems and current deficiency of universal regulatory guidelines, it could be appropriate for building managers and end-users to stay instructed apropos existing solution options, even as they may explore single-vendor closed-loop solutions. This survey assists in this mandate, assisting building owners and operators reach wise choices with both present and future matters in mind.

## 8. Conclusions

This paper discussed various recent technologies in the IoT, with a special emphasis on increasing energy effectiveness in buildings. The considerable number of analyses and reviews evaluated in this survey disclosed in what way the IoT could definitely end up serving as a powerful system for enhancing energy management in residential, commercial and other buildings, while pinpointing surmounting issues, which need to be more investigated and addressed as to suitably diffuse IoT in an effective manner. Moreover, this survey grouped the applications into five main types, which were energy consumption monitoring and control, predictive temperature control, occupancy and comfort sensing, controllable devices and the smart home application. In each case, literature was summarised that showed merit to each application. In addition to this, two case studies were presented from the literature that reported the energy savings after IoT implementation.

The main conclusions in this review are along the following lines:

- A number of researchers have confirmed the feasibility of applying IoT architectures for the problem of building management. Each of the building processes, such as HVAC, lighting, water circulation, etc., are capable of remote actuation and are shown to realise significant and quantifiable benefits from such IoT solutions.
- Given the sheer volume of sensors that are now dispersed across a building, it is obvious that the powering of sensors is complex. Researchers have explored a number of powering options from more conventional power over ethernet, to extracting renewable energy at a micro-scale. Other researchers have also explored reducing power requirements so traditional, standalone battery sources may become less cumbersome to manage. Through the remote wireless and low power constraints, it is recommended to use sensors with few data processing capabilities; complicated computations and postprocessing might be postponed to controllers with an extra stationary power supply.
- Both predictive and adaptive approached might be required to optimise energy efficiencies. Predictive methods are crucial to get a greater accurate profile of representative energy consumption, while adaptive methods are entailed for edge situations wherein the estimated energy pattern is imprecise within a given boundary.
- The good news is that a number of established communications protocols remain applicable for Building IoT systems (for example, Bluetooth, LoRaWAN (Long-Range long-power Wide Area Network), Wi-Fi, ZigBee or Z-Wave). More recent models have explored tiered architectures so clusters of sensors can use lower bandwidth and lower power to communicate among each other, but connectivity to central gateways would be of a higher bandwidth, from where high bandwidth solutions transmit information to applications in the cloud. Tiered network architectures become especially as Building IoTs find into wide-area solutions such as for Smart Cities.
- It was also shown that there are active challenges being addressed in the IoT community that are hindering the large-scale rollout of these systems. Primarily, the challenge is the security of the data that IoT systems collect and store. The trade-off that needs to be preserved amid private security and system effectiveness is set up based upon every single environment. The risk for harmful security infractions on a residential setting is significant. A very sophisticated security and encryption system can inhibit system effectiveness, as the computing capacities of the majority of small and isolated IoT platforms are in general limited. Newer technologies such as blockchain and machine learning offer significant promise and are also reviewed (along with more traditional security procedures and applications).
- Some of the better known as well as less obvious challenges that IoT systems face were raised. There are, as highlighted through the paper, several technical limitations that Building IoT systems need to overcome. Many deal with known trade-offs of power requirements, stability of connectivity, management of sensor deployments and security/privacy considerations. There are also critical business trade-offs that customers must make. End-to-end proprietary solutions by leading IT companies offer ease of use, rapid installations and access to “best-in-class” analytics. Yet, these benefits do come at a price, as customers lose ownership of their data and potentially face large switching costs in the future.
- There is not necessarily a “best” application for the IoT applied to building energy reduction. This is because each application is custom to building characteristics and location, as well as the goals of the application. For this reason, it is difficult to compare the cost and installation difficulty of an IoT application. However, if price and system complexity are not concerns, then each of the first four application types could be used together to maximise energy savings.

Suggested matters of more investigation comprise high performing and reliable security systems, powering of remote and design standards to warrant conformity through related uses. The more secure, customisable and safe the system hardware, software, networking, and security devices are throughout performing operations, the more achievable the technology advances will be.

Finally, IoT has been a promising solution with significant meaningful wins in industrial environments. Building IoT offers the promise to transform one of the largest business markets that is also primed for disruption. Over a third of all energy is consumed in buildings, making them a critical component of any global sustainability response. At the same time, buildings by nature of their high occupancy densities are central to security considerations including health securities that the pandemic has further brought into sharp focus. We believe that the next 5–7 years will see a massive transformation of buildings, with IoT solutions likely being one of the key enablers. This survey revealed many of the critical solution components are being successfully piloted in showcase environments. We expect it to be just a matter of time before they can be expanded to scale.

## Figures and Tables

**Table 1:** Summary list of research works on IoT infrastructures in building energy systems.

Authors	Publication Year	Topic	Focus
Ray et al. [7]	2018	Review	IoT architectures
Shakerighadi et al. [8]	2018	Review	IoT for modern energy systems
Pan et al. [9]	2015	Review	IoT framework for smart energy in buildings
Saleem et al. [10]	2019	Review	IoT-aided smart grid
Ray et al. [11]	2016	Review	IoT cloud platforms
Talari et al. [12]	2017	Review	Smart city-based on the IoT concept
Lin et al. [13]	2017	Review	IoT architecture, enabling technologies, security and privacy, and applications
Srinidhi et al. [14]	2019	Review	Network optimizations in the IoT
Dachyar et al. [15]	2019	Review	Knowledge growth and development in IoT research
Ghasempour et al. [16]	2019	Review	IoT in smart grid
Casini et al. [19]	2015	Sensors characteristics	IoT for energy efficiency of buildings
Wicaksono et al. [20]	2012	Sensors characteristics	Improving energy efficiency in building using ontology and building automation systems
Lork et al. [21]	2019	Sensors characteristics	An ontology-based framework for building energy management with IoT
King and Perry [22]	2017	Sensors characteristics Powering sensors	Smart buildings using IoT to save energy in existing buildings
Png et al. [23]	2019	Sensors characteristics	IoT for smart and scalable heating, ventilation and air conditioning control in commercial buildings
Prauzek et al. [24]	2018	Powering sensors	Energy harvesting sources, storage devices and system topologies for environmental wireless sensor networks
Gawali and Dehmukh [25]	2019	Powering sensors	Energy autonomy in IoT technologies
Curry and Harris [26]	2019	Powering sensors	Powering the environmental IoT
Matsui et al. [27]	2018	Predictive methods	Real-time sensing in residential area using IoT technology for finding usage patterns to suggest action plan to conserve energy
Marinakis et al. [28]	2017	Predictive methods	Decision support for intelligent energy management in buildings using the thermal comfort model
Mahdavinejad et al. [29]	2018	Predictive methods Adaptive methods	Machine learning for IoT data analysis
Mataloto et al. [30]	2019	Predictive methods	LoBEMS—IoT for building and energy management systems
Zou et al. [31]	2018	Predictive methods	Occupant activity driven smart buildings using WiFi-enabled IoT devices and deep learning
Lachhab et al. [32]	2018	Adaptive methods	Ventilation systems control using IoT and big data technologies
Fayaz and Kim [33]	2018	Adaptive methods	Energy consumption optimisation and user comfort management in residential buildings using a bat algorithm and fuzzy logic
Serra et al. [34]	2014	Architecture and networking	Smart HVAC control in IoT based on energy consumption minimisation with user comfort constraints
Kim et al. [35]	2017	Architecture and networking	Implementation of a low-cost energy and environment monitoring system based on a hybrid wireless sensor network
Mehta et al. [36]	2018	Architecture and networking	IoT applications and challenges
Sodhro et al. [37]	2019	Architecture and networking	Optimal resource management for IoT based green and sustainable smart cities
Pocero et al. [38]	2019	Energy consumption monitoring and control	Open source IoT meter devices for smart and energy-efficient school buildings
Mudaliar and Sivakumar [39]	2020	Energy consumption monitoring and control	IoT based real-time energy monitoring system using Raspberry Pi
Mihailescu et al. [40]	2020	Energy consumption monitoring and control	End-to-end anytime solution for appliance recognition based on high-resolution current sensing with few-shot learning
Santos and Ferreira [41]	2019	Energy consumption monitoring and control	IoT power monitoring system for smart environments
Hafeez et al. [42]	2020	Energy consumption monitoring and control	Efficient energy management of IoT-enabled smart homes under price-based demand response program in smart grid
Massano et al. [43]	2020	Energy consumption monitoring and control	An online grey-box model based on unscented Kalman filter to predict temperature profiles in smart buildings
Dell’Isola et al. [44]	2019	Energy consumption monitoring and control	An IoT-integrated tool to enhance user awareness on energy consumption in residential buildings
Luo et al. [45]	2019	Predictive temperature control	Development of an IoT-based big data platform for day-ahead prediction of building heating and cooling demands
Moreno et al. [46]	2017	Predictive temperature control	Applicability of big data techniques to smart cities deployments
Schmidt et al. [47]	2017	Predictive temperature control	Optimising legacy building operation based on the evolution into data-driven predictive cyber-physical systems
Al-Ali et al. [48]	2017	Predictive temperature control	A smart home energy management system using IoT and big data analytics approach
Wang et al. [49]	2018	Predictive temperature control	A dependable time series analytic framework for cyber-physical systems of IoT-based smart grid
Yassine et al. [50]	2019	Predictive temperature control	IoT big data analytics for smart homes with fog and cloud computing
Irshad et al. [51]	2020	Predictive temperature control	An IoT-based thermoelectric air management framework for smart building applications: A case study for tropical climate
Zhao et al. [52]	2020	Predictive temperature control	Intelligent thermal comfort controlling system for buildings based on IoT and AI
Lee and Yeo [53]	2020	Predictive temperature control	Condensation control to cope with occupancy activity and effectively mitigate condensation in unheated spaces by real-time sensor control strategy
Park et al. [54]	2020	Predictive temperature control	Reinforcement learning-based BEMS architecture for energy usage optimization
R. Carli et al. [55]	2020	Predictive temperature control	IoT-based architecture for model predictive control of HVAC systems in smart buildings,
Aliberti et al. [56]	2019	Predictive temperature control	A nonlinear autoregressive model for indoor air temperature predictions in smart buildings
Marinakis et al. [28]	2017	Occupancy and comfort sensing	Decision support for intelligent energy management in buildings using the thermal comfort model
Serra et al. [34]	2014	Occupancy and comfort sensing	Smart HVAC control in IoT based on energy consumption minimisation with user comfort constraints
Mahbub et al. [5]	2020	Controllable devices	IoT-cognizant cloud-assisted energy efficient embedded system for indoor intelligent lighting, air quality monitoring and ventilation
King and Perry [22]	2017	Controllable devices	Smart buildings using IoT to save energy in existing buildings
Hafeez et al. [42]	2020	Smart home application	Efficient energy management of IoT-enabled smart homes under price-based demand response program in smart grid
Tomat et al. [57]	2020	Occupancy and comfort sensing	Thermal comfort under the IoT Paradigm related to crowdsensing
Vanus et al. [58]	2019	Occupancy and comfort sensing	Prediction of CO_2_ course and occupancy recognition in intelligent buildings using IoT
Domb [59]	2019	Controllable devicesSmart home application	Smart home systems based on IoT
Rinaldi et al. [60]	2020	Controllable devices	A cognitive-driven building renovation for improving energy efficiency
Zigbee Alliance [61]	2020	Controllable devices	Project connected home over IP
Vanelli et al. [62]	2017	IoT data storage	IoT data storage infrastructure in the cloud using NoSQL databases
Chilipirea et al. [63]	2016	Smart home application	Smart home security system using IoT
Filho et al. [64]	2019	Smart home application WSAN in IoT	Energy-efficient smart home systems using IoT
Akyildiz et al. [65]	2010	WSAN in IoT	Wireless sensor networks
Chen et al. [66]	2010	WSAN in IoT	Distributed collaborative control for industrial automation with wireless sensor and actuator networks
Salarian et al. [67]	2012	WSAN in IoT	Coordination in wireless sensor-actuator networks
Villas et al. [68]	2013	WSAN in IoT	An energy-aware spatio-temporal correlation mechanism to perform efficient data collection in wireless sensor networks
Llaria et al. [69]	2016	WSAN in IoT	Application of wireless sensor and actuator networks to achieve intelligent microgrids: a promising approach towards a global smart grid deployment
Mocrii et al. [70]	2018	Smart home	IoT-based smart homes review
Mataloto et al. [30]	2019	Case studies of the IoT applied to building energy systems	LoBEMS—IoT for a kindergarten building and energy management systems
Han et al. [71]	2018	Case studies of the IoT applied to building energy systems	A case study in a university building for improving the energy saving process with high-resolution data and IoT
Brun et al. [72]	2018	Security	Deep learning with dense random neural network for detecting attacks against IoT-connected home environments
Andaloussi et al. [73]	2018	Security	Access control in IoT environment
Alkhalil and Ramadan [74]	2017	Security	IoT data provenance implementation challenges
Han et al. [75]	2019	Security	A novel architecture of air pollution measurement platform using 5G and blockchain for IoT applications
Dasgupta et al. [76]	2019	Security	Privacy of IoT-enabled smart home systems
Hasan et al. [77]	2019	Security	Attack and anomaly detection in IoT sensors in IoT sites using machine learning approaches
Casola et al. [78]	2019	Security	A security monitoring system for IoT
HaddadPajouh et al. [79]	2019	Security	IoT security
Mbarek and Pitner [80]	2018	Security	An efficient mutual authentication scheme for IoT
Pecorella et al. [81]	2018	Security	“Network Sentiment” framework to improve security and privacy for smart home

**Table 2:** Prospective energy reductions for diverse sensors.

System	Technology	Energy Reductions
Analytics	Cloud-based energy information system	5–10% whole building
Building automation	Building automation System	10–25% whole building
Lighting	Advanced lighting controls	45%
Lighting	Web-based lighting management system	20–30% over controls reductions
Window shading	Automated shade system	21–38%
Window shading	Smart glass	20–30%
Window shading	Switchable film	32–43%

**Table 3:** Power generation for different renewable energy sources [26].

Harvester	Power Output	Harvester	Power Output
Photovoltaic (Outdoor)	50 mW·cm^−2^	Air movement	6 W·cm^−2^
Photovoltaic (Indoor)	50 μW·cm^−2^	Pressure variation	15 μW·cm^−2^
Photosynthesis (Lab)	10–40 μW·cm^−2^	Piezoelectrics	12.5 μW·cm^−2^
Thermoelectrics	20 μW·cm^−2^	Triboeletrics	3 mW·cm^−2^
Pyroelectrics	8.64 μW·cm^−2^	Electrostatics	12 mW·cm^−2^
Microbial	3–700 μW·cm^−2^	Radio frequency	10.3 μW·cm^−2^
Chemical potential	3 mW	Induction	70+ μW·cm^−2^

**Table 4:** Outline of frequent IoT communication paradigms [41].

Wireless Technology	Data Rate	Max Charge Length	Communication Range	Security	Advantages
Bluetooth 5	125 kb/s (Long range S = 7) 2 Mb/s–500 kb/s (Long range S = 2)	255 Bytes	Up to 200 m +200 m (BLE)	L1–No security L2–AES 128 L3–AES and Pairing L4–ECDHE	Simple hardware; Easy access and operation; secure; low power consumption (BLE)
LoRaWAN	50 kb/s	243 Bytes	~5 km urban ~15 km–20 km rural	t AES	Availability; long communication range; secure; low power consumption; low cost
NB-IoT	200 kb/s	1600 Bytes	~1 km urban ~10 km rural	LTE encryption	wide maximum load length (4G coverage); secure
Sigfox	100 kb/s	12 Bytes	~10 km urban ~40 km rural	No Encryption or Adaptable for each case	Long communication range; low power consumption
Wi-Fi	Top 1 Gb/s IEEE 802.11ac	2034 bytes	1–100 m	WPA/WPA2	Advanced standard; high speed
ZigBee	20 kb/s @ 868 MHz 40 kb/s @ 915 MHz 250 kb/s @ 2.4 Ghz	255 Bytes	10–300 m Direct Line Sight 75–100 m Indoor	128 bit AES	Large number of nodes; low power consumption; low cost; secure
Z-Wave	100 kb/s	64 Bytes	~100 m (may vary depending on the number of nodes) (up to 4 hops)	Security 2 (S2) (Include AES-128, ECDHE, secure TLS tunnel)	Simple installation; Interoperability between devices of different manufacturers; low power consumption; secure

AES: Advanced encryption standard; BLE: Bluetooth low energy; ECDHE: Elliptic-curve Diffie–Hellman exchange; LTE: Long-term evolution; WPA: Wi-Fi protected access.

## Data Availability

Data are contained within this review article.

## Appendix A. Building Energy IoT Suppliers

A supplier survey was conducted to gain insight into the availability of building energy IoT products and services. As previously mentioned, there are various components of IoT systems and suppliers provide some or all of these elements. In Table A1 below, the list of suppliers investigated is provided. Each supplier is classified and sorted into providing one or multiple elements of an IoT system, titles scope. In order, the scope elements are sensors, controllers, smart devices, software, service and end-to-end. Controllers typically mean microcontrollers such as Raspberry Pi or Arduino. Service includes things like cloud storage or analytics. Last, end-to-end suggests a supplier that will handle every aspect of an IoT system installation. For each supplier, its name and potentially parent company are given, along with the tools the supplier uses and a general description. A link to the supplier website is included in each case.

**Table A1:** Building energy IoT supplier list.

Supplier	Reference	Parent Company	Scope	Tools	Description	Supplier Link
CONTEC	[83]	N/A	Sensors, controllers, Service	Source code, and IoT product supplier	Established IoT company with various applications and products, one of which focused on buildings and offices	https://www.contec.com/solutions/buildings-offices/ (accessed on 16 March 2021)
Lantronix	[84]	N/A	Sensors, controllers	Variety of microcontrollers and sensors	IoT product supplier with wide variety of product options	https://www.lantronix.com/ (accessed on 16 March 2021)
Particle	[85]	N/A	Sensors, controllers	Limited sensing tools with hardware	IoT hardware and cloud management company	https://www.particle.io/ (accessed on 16 March 2021)
SBT Alliance	[86]	N/A	Sensors, controllers, smart devices	lighting devices, sensors, and controllers	3-company alliance focused on improving energy efficiency largely through smart lighting	https://sbt-alliance.com/ (accessed on 16 March 2021)
sense	[87]	N/A	Sensors	Power meter in electrical panel	Smart meter that monitors usage and trends	https://sense.com/product/ (accessed on 16 March 2021)
Silicon Labs	[88]	N/A	Sensors, controllers	Microcontrollers, sensors, gateway connections	IoT company, sells products separately or provides solutions, tailored to building efficiency	https://www.silabs.com/ (accessed on 16 March 2021)
WebNMS	[89]	N/A	Sensors, service	Power meters, temperature/environment sensors, upload to server	Provides energy usage and temperature sensing inside and out to analyse trends and provide advice	https://www.webnms.com/iot/energy-management.html (accessed on 16 March 2021)
Amazon Echo	[90]	Amazon	Controllers	Smart speaker that can control other smart devices	Voice activated smart speaker that can interact with other smart devices	https://www.amazon.com/Amazon-Echo-Bluetooth-Speaker-with-Alexa-Black/dp/B00X4WHP5E (accessed on 16 March 2021)
Apple HomePod	[91]	Apple	Controllers	Smart speaker that can control other smart devices	Voice activated smart speaker that can control HomeKit devices	https://www.apple.com/ca/homepod/ (accessed on 16 March 2021)
Google Nest	[92]	Google	Controllers	Smart speaker that can control other smart devices	Voice activated smart speaker that can interact with other smart devices	https://store.google.com/ca/product/google_nest_mini (accessed on 16 March 2021)
HomeKit	[93]	Apple	Controllers	App that can control enabled smart devices	Homekit enabled devices can be controlled by apple electronics	https://www.apple.com/ca/ios/home/ (accessed on 16 March 2021)
Insight	[94]	Schneider Electric	Controllers	App that can control enabled smart devices	Energy management platform by Schneider Electric, Intended to go with their solar power system	https://solar.schneider-electric.com/product/insight/ (accessed on 16 March 2021)
Libelium	[95]	N/A	Controllers	Waspmote controller and gateway connection	IoT product creator, can provide solutions service or products separately	https://www.libelium.com/ (accessed on 16 March 2021)
Smart-Things Hub	[96]	Samsung	Controllers	App that can control enabled smart devices	Connects to a variety of smart devices to monitor and control from a smartphone app	https://www.smartthings.com/products/smartthings-hub (accessed on 16 March 2021)
Wink	[97]	N/A	Controllers, smart devices	App that can control enabled smart devices	Home monitoring, control and automation from one mobile application	https://www.wink.com/ (accessed on 16 March 2021)
GE Current	[98]	General Electric	Smart devices	LED lights, sensors, controls and analytics	Installs LED lights and controls them in a smart way using sensors and analytics	https://www.gecurrent.com/approach (accessed on 16 March 2021)
ecobee	[99]	N/A	Smart devices	Smart thermostat	Smart thermostat that senses activity, important rooms, grid demand and cost to optimise efficiency of heating	https://www.ecobee.com/en-ca/smart-thermostats/smart-wifi-thermostat-with-voice-control/ (accessed on 16 March 2021)
Lenovo Smart Home	[100]	Lenovo	Smart devices	Specific devices controlled with a smartphone app	A series of devices that can be controlled by smartphone app for convenience and comfort	https://www.lenovo.com/ca/en/smart-home/ (accessed on 16 March 2021)
Philips Hue	[101]	Phillips	Smart devices	Smart lighting fixtures, control app	Smart Home lighting—scheduled and smart phone controlled	https://www.philips-hue.com/en-us (accessed on 16 March 2021)
AT&T	[102]	N/A	Software	C++ Code for various microcontrollers	IoT software platform that connects other IoT systems	https://iotplatform.att.com/ (accessed on 16 March 2021)
AWS IoT	[103]	Amazon	Software	Code for various microcontrollers	Provides tools and solutions to connect IoT devices using microcontrollers	https://aws.amazon.com/iot/ (accessed on 16 March 2021)
Domoticz	[104]	N/A	Software	C++ Code for Raspberry Pi or Computer as controller	Open source software that allows monitoring and control of various devices though mobile or desktop devices	https://www.domoticz.com/ (accessed on 16 March 2021)
Home Assistant	[105]	N/A	Software	Python code for Raspberry Pi as controller	The biggest open source IoT platform. Monitor control and automate smart home processes	https://www.home-assistant.io/ (accessed on 16 March 2021)
openHAB	[106]	N/A	Software	Java code for Raspberry Pi or computer as controller	Open source software that connect various smart home systems, monitor and automate from an app or website	https://www.openhab.org/ (accessed on 16 March 2021)
Azure IoT	[107]	Microsoft	Service	Cloud connectivity, security	Detailed IoT management service	https://azure.microsoft.com/en-us/overview/iot/ (accessed on 16 March 2021)
Cisco	[108]	N/A	Service	Data analytics and storage	Provides IoT service including networking, data management and security	https://www.cisco.com/c/en_ca/solutions/internet-of-things/overview.html (accessed on 16 March 2021)
Ericson	[109]	N/A	Service	Data analytics and storage	IoT and connectivity and analytics company for industrial applications	https://www.ericsson.com/en/internet-of-things (accessed on 16 March 2021)
Oracle	[110]	N/A	Service	Data analytics and storage	Analytics and cloud monitoring company for industrial applications	https://www.oracle.com/ca-en/internet-of-things/what-is-iot.html (accessed on 16 March 2021)
Avnet	[111]	N/A	End-to-end	Tools for every step of an IoT solution	End-to-End IoT company for companies	https://www.avnet.com/wps/portal/us/solutions/iot/ (accessed on 16 March 2021)
Vates	[112]	N/A	End-to-end	Tools for every step of an IoT solution	End-to-End IoT solutions in wide variety of applications	https://software.vates.com/internet-of-things/ (accessed on 16 March 2021)
